# Understanding Binding of Chitosan to Graphene in Li–Ion Battery Anodes from First-Principles

**DOI:** 10.1021/acsaem.5c03926

**Published:** 2026-02-04

**Authors:** Burak Ozdemir, Rita Magri

**Affiliations:** † Dipartimento di Scienze Fisiche, Informatiche e Matematiche, Università di Modena e Reggio Emilia, Via Campi 213/A, 41125 Modena, Italy; ‡ Centro S3, Istituto Nanoscienze-Consiglio Nazionale delle Ricerche (CNR-NANO), Via Campi 213/A, 41125 Modena, Italy; § Centro Interdipartimentale di Ricerca e per i Servizi nel settore della produzione, stoccaggio ed utilizzo dell’Idrogeno H2-MO.RE., Via Università 4, 41121 Modena, Italy

**Keywords:** chitosan, organic polymer binder, Li–ion battery, graphite anode, graphene, DFT

## Abstract

As a water-soluble, biodegradable, and abundant biopolymer, chitosan presents great advantages over the common PVDF as a possible binder to be used in Li–ion battery graphite anodes. Using accurate density functional theory, which includes a van der Waals long-range energy functional, we have determined that chitosan molecules physisorb on graphene/graphite and orient themselves horizontally, exposing the hydrogen atoms and the amino group to the surface. The binding energy is less than half that of PVDF. The binding is accompanied by the transfer of 0.021 e from graphene to chitosan and is dominated by the van der Waals long-range interactions. We have then investigated how the functionalization of graphene using point defects, oxygen atoms, and OH and LiF molecules as adsorbates affects the binding properties of chitosan. We have found that the presence of carbon vacancies and the functionalization with the OH and LiF molecular adsorbates are effective at increasing the binding. Room temperature significantly increases the chitosan binding energy on graphene functionalized with atomic oxygen or OH groups. The interaction with charged ions in low and high pH environments strongly increases chitosan adhesion to graphene, to which the charge is transferred. The changes of the binding properties of chitosan to the different surfaces are monitored through the analysis of the electronic charge redistribution, Bader charges, and changes in the electronic structure. Our results constitute a baseline study for further investigations of the interaction between the eco-friendly polymeric chitosan and the active material surfaces of electrodes in energy devices.

## Graphical abstract

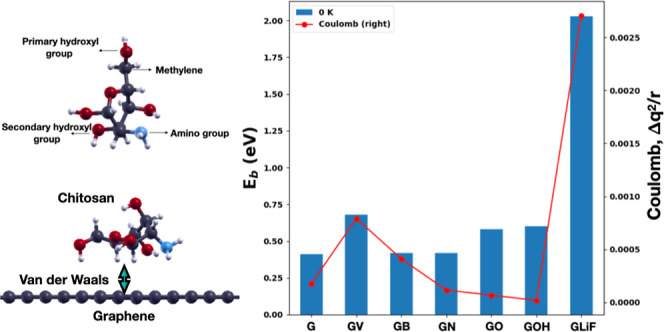

## 1 Introduction

Due to accelerating climate change, the development and optimization of clean energy technologies and batteries has become a major research priority. Although increasing battery capacity—for example, by using silicon anodes—is important, other components such as the binder also require significant improvement.^1–3^ In the conventional Li–ion battery graphite anode, the polymeric binder used to hold the particles together and improve adhesion to collector and conductive additives is polyvinylidene fluoride (PVDF). However, PVDF has several disadvantages, such as weak adhesion to electrode particles, unwanted reactions with lithiated graphite at elevated temperatures that form LiF, low Li^+^ diffusion at high C rates that hamper Li intercalation, which results in the deposition of the Li metal on the surface of the active material, high cost, and the requirement of toxic solvents in its production. The *N*-methyl-2-pyrrolidone (NMP) solvent residues can cause cracks during the battery charge/discharge, which decrease the capacity and the lifetime of the battery.^4^ α-PVDF is the most easily obtained form and is used as a binder in battery electrodes.^5^ According to the review of Wang et al.,^4^ a good binder should possess the following properties: (i) a wide working temperature and the capacity to maintain the strength of the binding force; (ii) good mechanical properties including tensile strength, elasticity, flexibility, and adhesive strength under tension or compression conditions, in order to withstand large volume changes or a strain change particularly in high capacity electrodes such as silicon (Si) anode; (iii) resistance to electrolyte swelling; (iv) promotion of the generation of stable cathode electrolyte interphase (CEI)/solid electrolyte interphase (SEI) layers; (v) adequate electrical and ionic conductivity for an excellent electrochemical performance; (vi) good dispersion properties in solvent in order to obtain homogeneous mixtures with the active material particles; (vii) outstanding chemical and electrochemical stability in different chemical solvents even under a high voltage window; and (viii) a low-cost, environmentally friendly, and facile preparation.

Chitosan, (C_6_H_13_O_5_N), is derived from chitin, which is the second most naturally abundant biopolymer and is present in crustaceans, mollusks, insects, and certain fungi. Due to its abundance in nature, chitosan is a low-cost material. Its price can be as low as 5$/kg, whereas PVDF has a price of 19–23 $/kg.^6^ Because of its biocompatibility and biodegradability, it has a potentially wide range of applications such as water treatment, separation membranes, food packaging, tissue engineering, drug delivery, and batteries. It dissolves in nontoxic dilute aqueous acid solutions, making it an ecofriendly material.^7^ Therefore, it meets several of the criteria outlined above for an effective binder: it is low cost, disperses well in eco-friendly acidic solvents, is easy to prepare, and provides good mechanical properties for battery applications.

The monomer has three reactive functional groups: an amino group, primary and secondary hydroxyl groups, as represented in Figure 1, where the amino group has the greatest influence on the structural and physicochemical properties of the polymer^8^ due to its lithiophilicity. The first applications of chitosan in batteries have been as a natural solid-state electrolyte. A chitosan–Zn electrolyte has been recently used in Zn metal batteries showing a good Zn^2+^ ion conductivity.^9^ Also, Kim et al. used reduced graphene oxide–chitosan composites as binders in Li–S batteries and showed an improved battery lifetime.^10^ In different experimental papers, it is mentioned that chitosan-based binders used for graphite anodes, or Si, Si/graphite composite anodes, have high ionic conductivity, mechanically robust behavior, better Coulombic efficiency, and capacity retention. Based on that literature, there is a significant difference in the mechanical flexibility and durability of chitosan-based binders compared to traditional PVDF binders, particularly under cycling. Chitosan-based binders offer superior mechanical stability and adhesion to electrode materials compared to PVDF, especially for anodes undergoing large volume changes.^11–14^ Another experimental study investigated the usage of chitosan-based binders in electrochemical double-layer supercapacitors.^15,16^ First-principles and molecular dynamics (MD) calculations of chitosan for drug delivery have also been reported.^17–19^ The MD study of Zahra et al. shows that pure graphene–chitosan has a larger diffusion coefficient of cyclophosphamide, an anticancer drug, relative to N- and P-functionalized graphene–chitosan. In a recent study, using first-principles calculations, reactive active sites have been determined for a chitosan–KOH electrode for lithium–sulfur batteries using first-principles calculations.^20^ The pharmaceutical application of chitosan-functionalized graphene has also been reported.^21^ In a density functional theory (DFT) study concerning removal of Mn and V from wastewater, chitosan adsorption on graphene oxide has been studied and chitosan adsorption energy on graphene oxide is calculated as 0.53 eV.^22^

**Figure 1:**
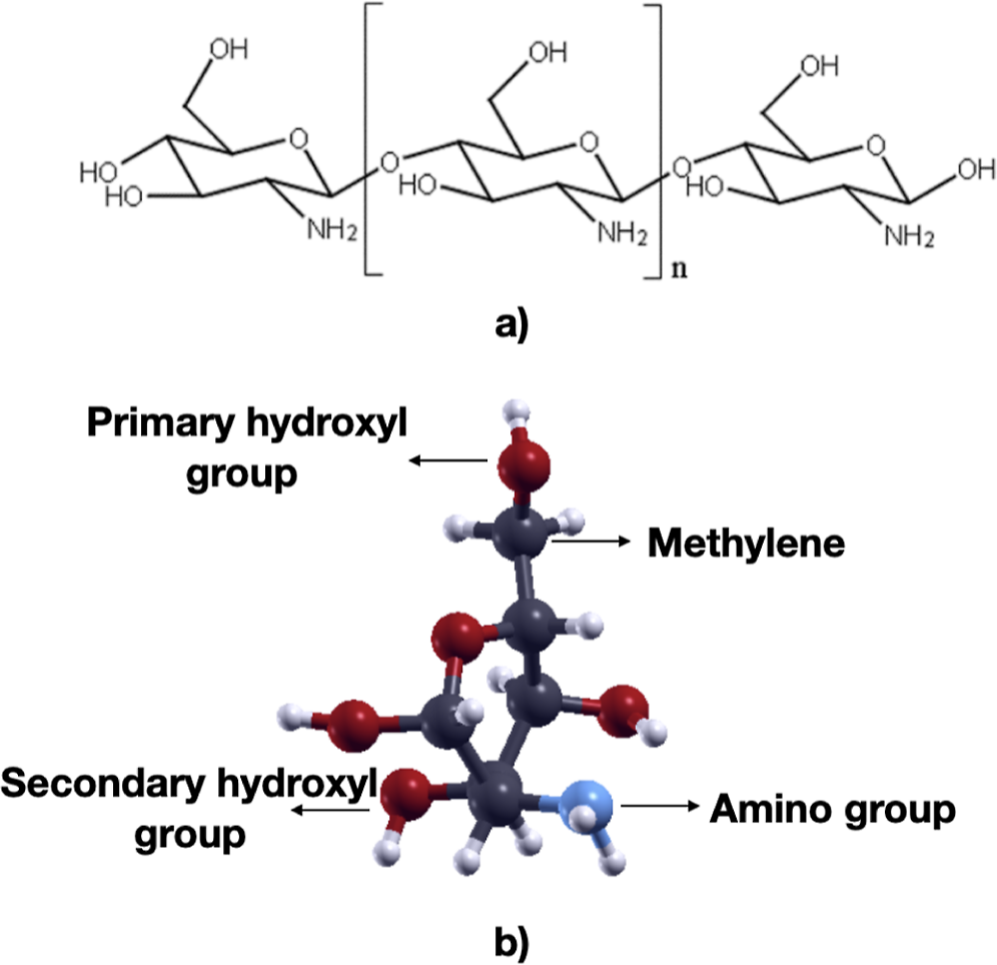
(a) Skeletal representation of chitosan monomer formula; (b) ball–stick model of chitosan where the 3 functional groups are shown. Color codes: C: dark gray, O: red, N: blue, H: small white.

The mechanical properties of chitosan by itself are not optimal for applications, such as tissue engineering, organic conductive films, and heat-resistant material, where mechanical strength is required; therefore, for these applications, composites of chitosan and graphene oxide have been extensively studied.^23^ Experimentally, it has been shown that graphene oxide can disperse well in chitosan and the mechanical strength increases compared to pure chitosan.^23,24^ It is reported that the H atoms belonging to C–O and amino groups of chitosan bind strongly to the functional groups of graphene oxide, and also the ductility of chitosan–graphene oxide nanocomposites is higher compared to pristine chitosan, with 2 wt % GO in chitosan showing the best mechanical properties.^25^ From Fourier transform infrared (FT-IR) spectra, it is understood that the interaction between chitosan and graphene oxide is through H atoms.^26^ In another experimental study, it is shown that the strong interaction between GO (and rGO) and chitosan is mediated by the carboxylated groups of GO and the NH_3_^+^ ions of chitosan.^27^ Also in the same study, the tensile strength and toughness of the GO–chitosan assembly are shown to improve.

Few studies have investigated chitosan interaction with pristine, defected, and functionalized graphene, of which we give here an overview. The binding energy of chitosan to pristine graphene was calculated in the range of 0.04–0.05 eV by Zhang et al.^28^ COOH^–^ was found to lead to a stronger chemical binding between chitosan and graphene, in particular when the amino group was exposed to COOH^–^. OH^–^ functionalization was shown to improve the binding energies to a lesser extent, and the Stone–Wales defect was found to be not very influential on the binding of chitosan.^28,29^ Ebrahimi et al.^29^ calculated the binding energy of chitosan to graphene and found it to be 0.63 eV using a revPBE exchange correlation (xc) functional that includes the Grimme dispersion correction with the Becke–Johnson damping (D3BJ). In that study, the graphene sheet was modeled with a nanosheet of 96 carbon atoms passivated on the border by hydrogen atoms. Adsorption of chitosan to carbon nanotubes has been studied from first-principles calculations using the PBE exchange–correlation functional, finding an adsorption energy of 0.22 eV.^30^ The ionic conductivity of protons (through hydronium ions) of chitosan–graphene oxide composite systems has been studied using MD simulations as a function of the water content, the pH level (controlled by the protonation degree of the amino groups of chitosan), and temperature, finding that a 40% water content is the most suitable and the temperature and pH levels are important factors in proton diffusion.^31^ Another MD study also investigated the effect of pH levels by controlling the protonation and found that a basic environment results in the aggregation of the chitosan chains, enhancing the encapsulation of graphene quantum dots.^32^ In a DFT study, the binding of the NH_2_ group of chitosan with the carboxyl-terminated edges of GO is modeled and studied to investigate its suitability for dopamine sensor applications.^33^ Binding of OH to the amino group of chitosan is studied with DFT, and it is found that the central monomers are energetically more favorable than terminal monomers.^34^

As for the structural studies, an MD study revealed two different conformations of chitosan, one extended, crystalline-like, and the other hairpin-shaped, very close in energy but separated by a high energy barrier.^35^ Bulk properties of chitosan ensembles such as the *V*–*T* curve, glass transition temperature, and tensile modulus were determined by MD simulations and found in good agreement with previous experimental data.^36^ The dependence of the effective charge state of chitosan on pH and temperature has been studied experimentally in the work of Lupa et al. where it was found that the electrophoretic mobility of the chitosan molecules becomes maximum at pH 2 and decreases toward higher pH levels.^37^

Because of their many advantages, chitosan-based binders have been recently proposed. Studies specifically targeting this application are scarce, in particular those focusing on the material modeling and simulations, that would be much needed for the design of improved and electrode-specific binders. This paper fills in the gap exploring the adhesion (binding) properties of chitosan to a graphene/graphite electrode and compares them to those of the most commonly used PVDF binders. The study is carried out using highly accurate DFT computations. Although the effect of the Stone–Wales defect and functionalization of graphene on chitosan binding have already been modeled using first-principles approaches, the effects on the binding energy resulting from other defects such as isolated C vacancies and substitutional boron and nitrogen atoms have never been studied before as well as those due to lithiation and higher temperatures. To simulate the physical situations that can occur at the graphite anode of a lithium ion battery, we have considered not only the different modifications and functionalizations of the graphene sheet but also (i) the interaction with the electrolyte salt molecules; (ii) the presence of lithium ions; and (iii) the role of pH and temperature. The ab initio description of the binder/electrode interaction is a very challenging task. To understand the behavior of this complex system, a simpler model is necessary, so we address here the interaction of a single chitosan monomer with the electrode surface described using the slab method. In this work, our primary objective is to establish an atomistic understanding of the adhesion mechanisms of chitosan on graphene/graphite anodes by using first-principles calculations. We test the hypotheses that (i) chitosan binds to pristine graphene predominantly through van der Waals interactions with minimal electronic hybridization; (ii) defects, surface functionalization, and electrolyte-derived species can significantly enhance chitosan adhesion; and (iii) environmental factors relevant to battery operation—such as lithiation, pH, and temperature—strongly modulate binding strength and charge transfer. In Section 3.1, we first establish the baseline interaction mechanism between chitosan and pristine graphene to identify the dominant binding contributions. In Section 3.2, we then examine how realistic graphene modifications alter chitosan adhesion, directly testing the tunability hypothesis and also the van der Waals nature of the interaction lacking electronic hybridization. In Sections 3.3, Sections 3.4, and Sections 3.5, we answer the question of how environmental factors affect the binding of chitosan. The results of this investigation can then be applied to the analysis and design of more complex chitosan-based materials.

## 2 Method

Calculations based on DFT are carried out using the Quantum-Espresso software suite^38–40^ The vdW-DF2-C09 ^41–47^ exchange–correlation (xc) functional with the van der Waals correction and norm-conserving pseudopotentials^48^ are used. We have set an 80 Ry kinetic energy cutoff on the energy of the plane waves. Charge integration was obtained using a 12 × 12 × 1 Monkhorst–Pack *k*-point grid for pure graphene and 2 × 2 × 1 *k*-point grid for the graphene–chitosan/PVDF cell. This last cell was constructed by taking the $3\sqrt{3}\times 3\sqrt{3}\times$ 1 graphene cell (in order to be able to also model chitosan interaction with LiC_6_) and inserting the chitosan or PVDF molecules inside. The in-plane distances between chitosan and its replicas for this supercell dimension are 6.60 and 8.45 Å. Isolated chitosan molecule is modeled inside a box with dimensions of 10 × 10 Å × 10 Å. Chitosan adsorption on graphene surfaces is modeled with an 18 Å vacuum spacing. We set the self-consistent field energy convergence threshold at 1 × 10^–6^ Ry using the Marzari–Vanderbilt smearing method with a 1 × 10^–4^ Ry Gaussian spreading.^49^ The final energy of the optimized structures was converged better than 1 meV/atom with respect to the kinetic energy cutoff and *k*-point sampling. A 10 × 10 × 1 *k*-point grid is used for the density of states calculation of graphene–chitosan systems. The binding energy of the chitosan/PVDF molecule to graphene is calculated according to the formula

1 $$E_{b}=-\left[E\left(G+molecule\right)-E\left(G\right)-E\left(molecule\right)\right]$$

where *E*(G + molecule) is the total ground-state energy of the system comprising graphene (G) and the adsorbed molecule (either chitosan or PVDF).

In order to assess the reliability of our methodology, we calculated the binding energy of benzene on pure graphene and obtained a difference of 5 meV/atom compared to the same result in the literature,^50^ which can be explained due to differences in the computational parameters and pseudopotentials. To check if this absolute error can affect the relation between binding energies for benzene adsorption on different graphene-based systems, we also calculated the binding energy of benzene on a single carbon vacancy in graphene. In this last case, we found that the binding energy is smaller, in agreement with the published paper’s result, with a similar error of 6 meV/atom. Thus, despite the difference in the calculation parameters, the obtained physical picture is the same. Also, the atomic structures compare well (see the Supporting Information). The vdW-DF2-C09 functional was chosen because long-range dispersion interactions dominate polymer–graphene adhesion^51^ and are poorly described by semilocal functionals. Benchmark calculations for benzene adsorption on graphene reproduce literature values within a few meV per atom, validating the accuracy of the present setup.

Due to dynamic nature of the charge transfers^52^ and also the importance of electric dipoles in the interaction between graphene and polymers^53^ here in this work, we employed ab initio molecular dynamics calculations (AIMD). In the AIMD simulations, a 1 × 1 × 1 cubic cell with a 15 Å lattice constant is used for the isolated chitosan molecule. A 2 × 2 × 1 cell (w.r.t. the cell of chitosan adsorbed on graphene), which includes only one chitosan monomer (the other 3 chitosan monomers are deleted), is used for modeling chitosan on graphene. In these calculations, variable cell calculations are performed at zero pressure. A time step is taken as 1/10th of the oscillation period of the hydrogen molecule at room temperature, which is 0.79364 fs and the simulations are run for 3 ps. The temperature-dependent binding energies are calculated according to the following formula

2 $$E_{tot}\left(A,T\right)=E_{g}+E_{K}\left(atoms,T\right)$$

3 $$E_{b}\left(T\right)=\left\langle E_{b}\right\rangle av$$

4 $$=\langle -{\left[E_{tot}\left(G+molecule,T\right)-E_{tot}\left(G,T\right)-E_{tot}\left(molecule,T\right)\right]\rangle }_{av}$$

where *E*_tot_(*A*, *T*) is the energy of system *A* at temperature *T*, which is the sum of the energy of the ground state, *E*_g_, and of the kinetic energy of the atoms, *E*_K_(atoms, *T*). The binding energy is calculated according to eq 1, taking *E*_K_ (atoms, *T*) also into account. Finally, the average binding energy over the simulation time is calculated.

For multilayer graphene, we define the interlayer binding energy as follows

5 $$E_{b}=-\left[E\left(MG\right)-NL\times E\left(Graphene\right)\right]/NA$$

where *E*(MG) is the total energy of the multilayer graphene with NL being the number of layers, *E*(Graphene) is the energy of a single layer graphene, and NA is the total number of atoms.

For modeling the effects of the environmental pH levels on chitosan, the chitosan molecule has been charged by adding to it charged ions (OH^–^ for the basic environment, H^+^ in place of H for the acidic environment). Specifically, for the acidic environment, one of the H atoms belonging to the amino group is charged as +1, while to model a high pH level, one of the H atoms of the amino group is replaced by an OH^–^ group with a starting charge of −1 on the O atom. The code automatically adds or subtracts the atomic electron charge density at the proper location of the ion when the starting charge distribution. To be able to converge the calculations, a neutral unit cell is required so a compensating uniform background charge is added.

The charged system calculations can be affected by errors due to spurious electrostatic interactions between periodic replicas and between the localized charges and the uniform charge background, so the results have been corrected by repeating the calculations using increasingly larger supercells and obtaining the converged binding energies at infinity by a fit to a model function; *E*_b_ = *a* + *b*/*L* + *c*/*L* ^3^, where *a*, *b*, and *c* are the constants to fit and *L* is the lattice constant.^54^

The Bader charge analysis is carried out using the Critic2 software.^55,56^ The van der Waals energy (nonlocal correlation) is calculated with the postprocessing code ppacf.x distributed by Quantum-Espresso software.

The local density of states (LDOS) were calculated, summing the partial densities of states over atoms and orbitals for the system (chitosan and graphene) in which more than one atom of the same kind is present.

## 3 Results and Discussion

### 3.1 Interaction of Chitosan with a Pure Graphene Sheet

We first established the baseline interaction mechanism between chitosan and pristine graphene to identify the dominant binding contributions. To study the interaction between the chitosan (and PVDF) molecule and the graphene sheet, six different relative orientations were considered, obtained as shown in Figure 2, where the graphene sheet orientations correspond to the planes indicated by the numbers from 1 to 6. Due to the symmetry of PVDF, only five orientations were considered for PVDF. These orientations are referred to in the following as Ori.1, Ori.2,···, Ori.6. The initial configurations for force optimization were built by lowering the molecules toward the graphene sheet, so that the distance of the closest atom of chitosan to graphene is 1 Å. A longer initial distance of about 2.4 Å was also considered.

**Figure 2:**
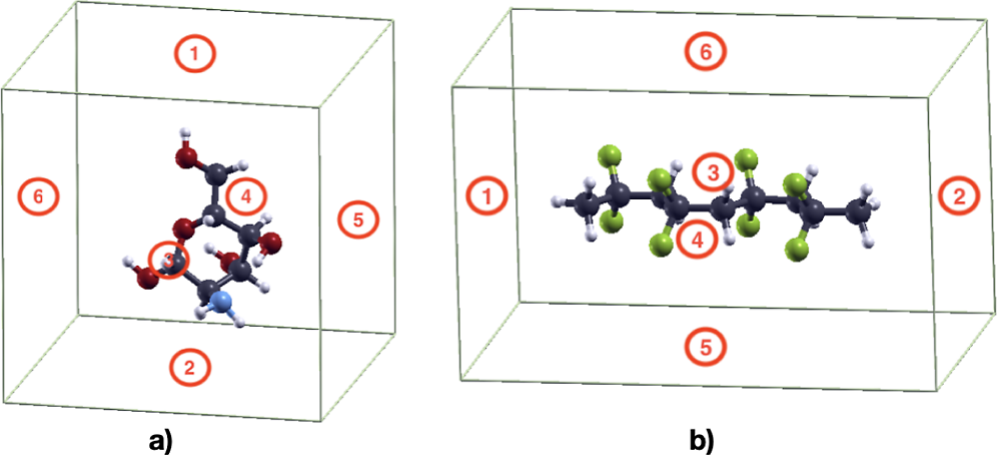
Orientations of the graphene layer relative to (a) chitosan and (b) PVDF molecules are represented with red circles. Color codes: C: dark gray, O: red, N: blue, H: small white.

The optimized structures, binding energies, and final distances of the closest atom of chitosan to the graphene surface are shown in Figure 3. Interestingly, the lowest energy distances are longer than the initial one and are on the order of 2.4–2.6 Å. The strongest binding configuration for chitosan is Ori.3 with a binding energy of 0.41 eV. This value is significantly higher than the 0.04–0.05 eV reported in the work of Zhang et al.,^28^ because in that study the PBE functional does not include the van der Waals interaction contrary to ours which accounts for most of the binding. The configuration with the highest binding energy has the largest number of H atoms closer to the graphene sheet. We can also observe that the distance of the amino group from graphene affects the strength of the interaction since the energetically most favored orientations are Ori.3, Ori.6, and Ori.2, which have the closest distances of the amino group from graphene. We also calculated the dependence of the binding energy of Ori.3 on the in-plane angles relative to the graphene sheet, finding that the maximum energy variation is only 0.013 eV. A Bader charge analysis indicates a net charge transfer of 0.021 e from graphene to chitosan. We can compare our result with other values reported in the literature. A charge transfer of 0.12 e from graphene to chitosan was calculated within a localized basis set and the revPBE xc functional approach and using the Hirshfeld method.^29^ The larger charge transfer can be explained by the fact that in that work, the graphene sheet was described using a nanosheet with 96 C atoms that can more easily deform and move toward the chitosan monomer, increasing the charge transfer. Furthermore, the configuration of the chitosan monomer on graphene is different. A net charge transfer of 0.064 *e* between chitosan and a carbon nanotube has also been reported.^30^ These values are in reasonable agreement with those obtained by us.

**Figure 3:**
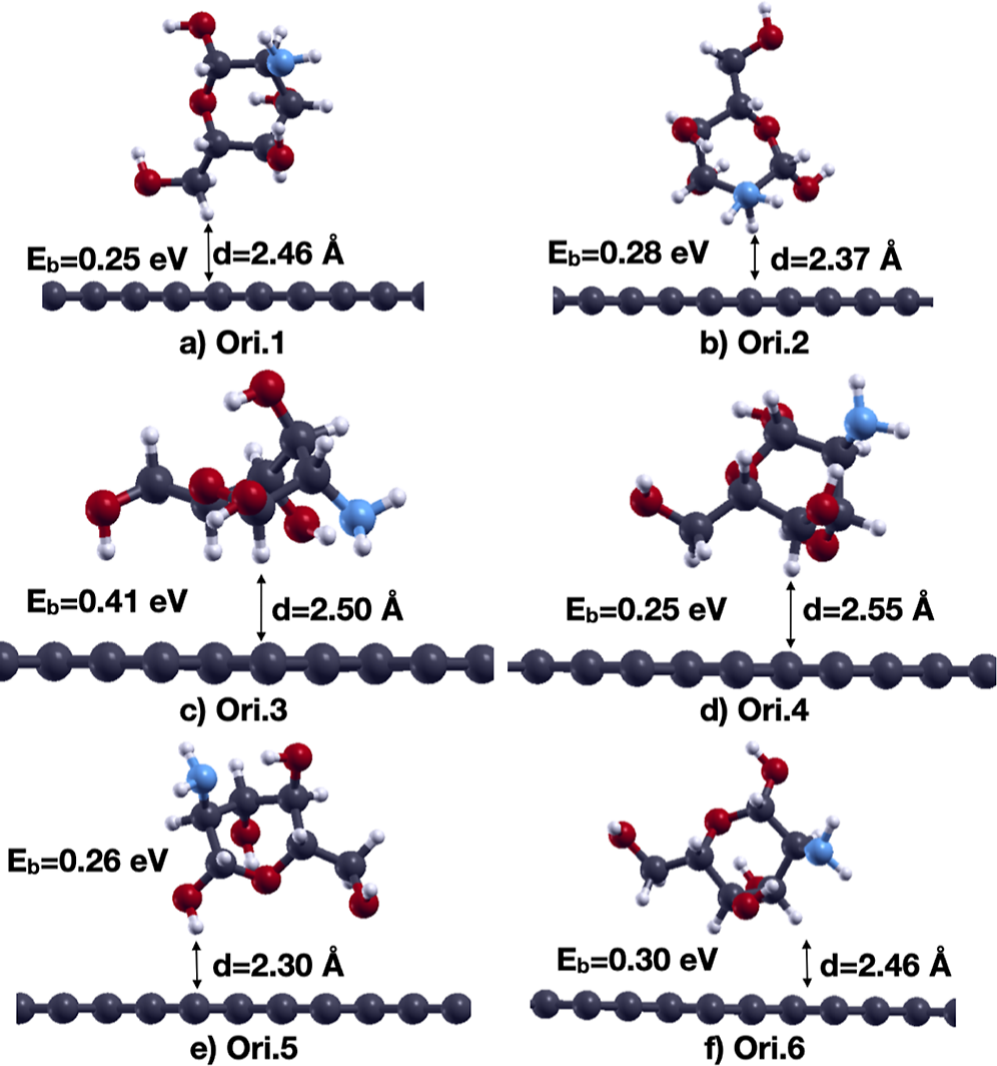
Binding energies (*E*_b_) and graphene–chitosan distances (d) of 6 different orientations (a–f) of chitosan on pristine graphene determined as shown in Figure 2. Color codes: C: dark gray, O: red, N: blue, H: small white.

We plotted the charge density redistribution due to the adsorption of the chitosan monomer on graphene as the difference between the charge density of the chitosan/graphene system and the charge densities of the isolated chitosan and graphene. The charge density difference is shown in Figure 4. In the figure, we see that the H atoms of chitosan closer to the graphene sheet lose electrons. Interestingly, the presence of the chitosan monomer on the graphene sheet leads to separation of the graphene electronic charge in different regions of charge accumulation and depletion. The charge depletion region on graphene is under the terminal group of chitosan. This region is responsible for the net charge transfer from graphene to chitosan. The terminal group is where the chitosan monomers connect to form a polymeric chain. The charge accumulation regions on graphene are in close proximity to the amino group, the primary hydroxyl group, and the second terminal group, where the charge transfer is reversed, from chitosan to graphene. Moreover, these findings on the charge difference distribution are independent of the in-plane angle formed by the chitosan monomer on graphene. In Figure 4c, we show the Bader charge differences due to the exposure of chitosan to graphene. The main charge redistribution (more than 1 order of magnitude relative to all the others) in chitosan occurs along a nitrogen–hydrogen bond in the amino group (atoms 11 and 12). The electronic charge transfers from the nitrogen atom back to the hydrogen atom, indicating weakening of the bond. This bond weakening is due to the hydrogen atom interaction with the atoms of graphene. Due to the importance of the amino group in the modifications of chitosan, this result is notable.

**Figure 4:**
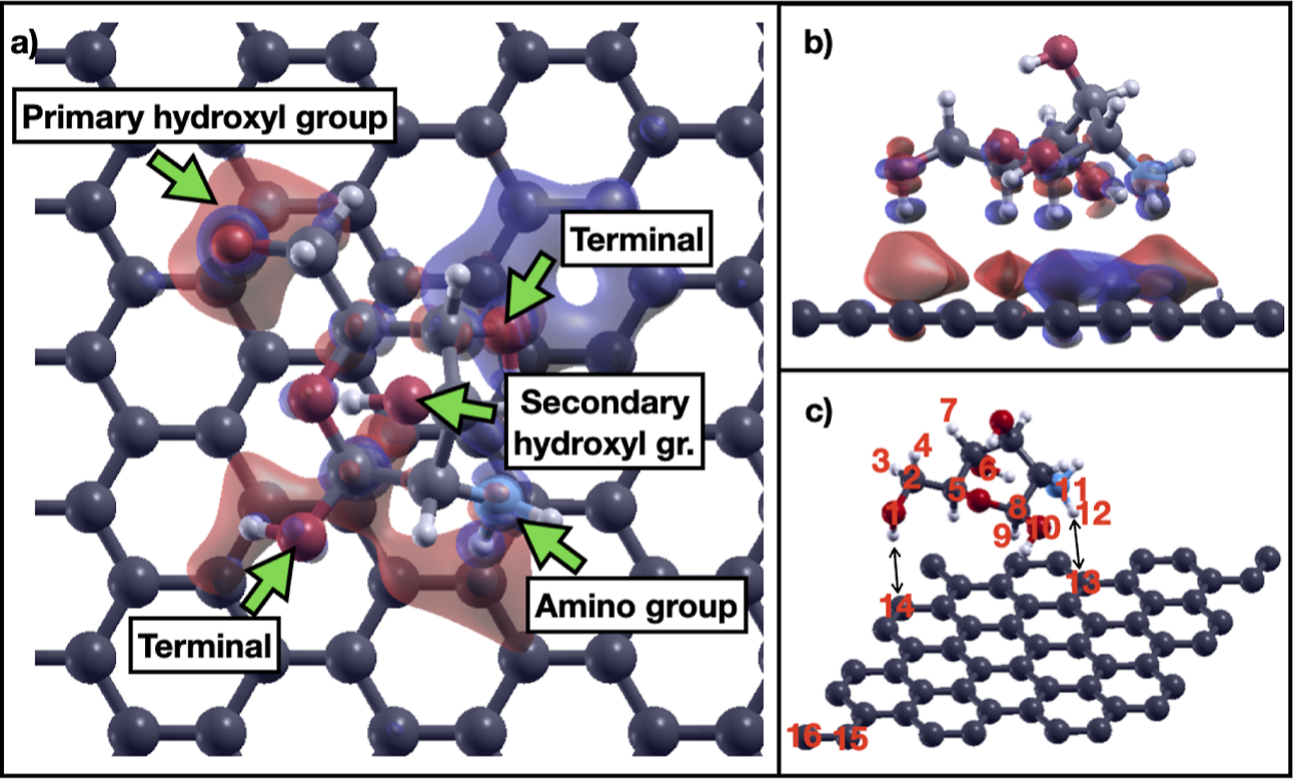
Charge density redistribution of chitosan on pristine graphene. “Terminal” refers to the ending groups of the chitosan chain where two chitosan monomers connect through the O atom, here saturated with a H atom. Isovalue for the charge density is 0.0003 (red: charge accumulation, blue: charge depletion). (a) Top view, (b) side view, (c) Bader charge differences of the chitosan/graphene system relative to isolated chitosan and graphene. The arrows indicate the closest C atoms of graphene to selected H atoms of chitosan. Red-colored numbers are the identification numbers of the atoms that have a relatively large charge difference. (1) O: −0.014, (2) C: +0.058, (3) H: +0.018, (4) H: −0.044, (5) C: +0.011, (6) O: +0.012, (7) H: −0.030, (8) C: −0.039, (9) H: +0.018, (10) O: +0.013, (11) N: +0.383, (12) H: −0.381, (13) C: −0.011, (14) C: −0.012, (15) C: +0.011, (16) C: +0.015, (17) C: −0.013. Color codes: C: black, O: red, N: blue, H: small white.

### 3.2 Defective or Functionalized Graphene and Comparison with PVDF

We then examine how realistic graphene modifications alter chitosan adhesion, directly testing the tunability hypothesis. We investigated how the presence of defects in the graphene sheet affects the binding energy of chitosan. The presence of single carbon vacancies in graphene is known from the literature^57^ Therefore, we studied the impact on the chitosan–graphene binding energies of isolated carbon vacancies (GV) and of point substitutional defects: boron (GB) and nitrogen (GN). We also studied the changes in the structures and binding energies of chitosan on graphene due to the presence on graphene of an additional oxygen atom (GO) and a hydroxyl group (GOH). Since a binder interacts with the electrolyte, we considered the presence on graphene of a solid-electrolyte-derived LiF (GLiF) and studied the induced changes in the interaction between chitosan and graphene. The optimized structures of the modified graphene and the corresponding charge distributions are given in Figure S1 in Supporting Information.

To study changes in binding energies due to defects and functional groups in graphene, we have adopted the Ori.3 configuration since we have found that chitosan binds more strongly to pure graphene in this orientation. The calculated binding energies of chitosan to modified graphene are shown in Figure 5. For GV, GB, and GN, we placed the defect under the amino group that is the most reactive part of chitosan.^8^ For GO, GOH, and GLiF, we found that chitosan is repelled if these functional groups are located under the amino group. Therefore, they were located under the chitosan primary hydroxyl group since in this case there was no repulsion. In the case of GLiF, the LiF molecule is initially positioned perpendicular to the graphene layer with the Li ion below. This configuration is the lowest energy one for LiF on graphene. We see that the substitution of a carbon atom of graphene by boron and nitrogen does not have significant effects on the binding energy, although the net transfer of charges from graphene to chitosan differs from that of pure graphene (Figure 5). This is because boron having only one electron in the p shell, one electron less than carbon, tends to lose it more easily (lower ionization potential) than nitrogen, which has three electrons in the p shell, one more than carbon (higher ionization potential). However, the charge density differences are similar (Figure 6). The binding energy of chitosan to the GV is larger. This is due to the smaller distance between chitosan and GV, 1.92 Å compared to 2.51 Å in pristine graphene, and to a larger transferred charge.

**Figure 5:**
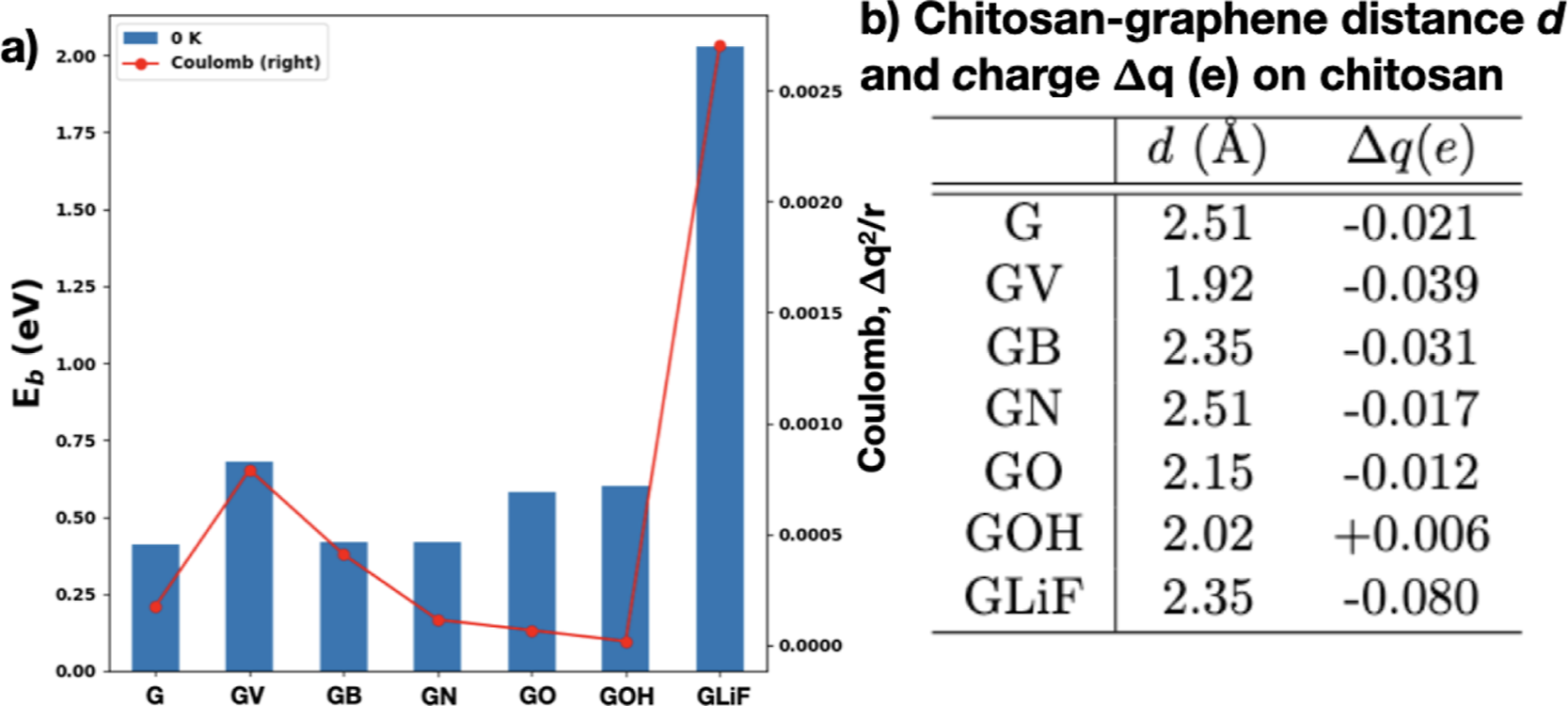
(a) Binding energies of chitosan to pure and defective graphene at 0 K and comparison with the simple Coulomb model explained in the text; (b) distances between the closest H atom of chitosan to graphene and charge transfer from graphene to chitosan calculated with a Bader charge analysis.

**Figure 6:**
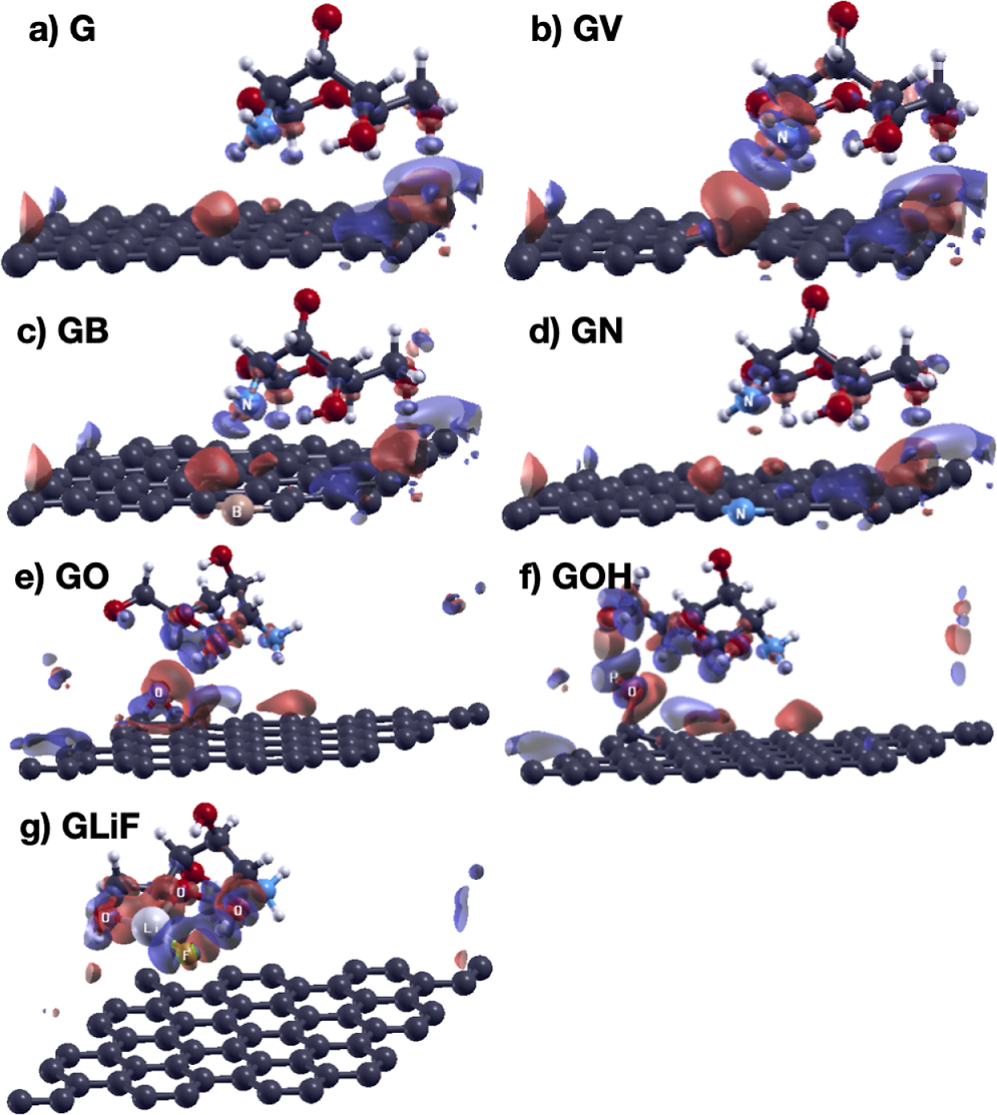
Charge difference distribution upon chitosan adsorption on pure and defective graphene (a–g). Red regions represent charge accumulation, and blue regions represent charge depletion. The isovalues are 0.002 for GLiF and 0.0005 for the rest. Color codes for the atoms: C: dark gray, N: blue, O: red, H: small white, B: orange, Li: large light gray, F: green.

Chitosan on GO and GOH binds more strongly than that on pure graphene. We observe that the distance between the oxygen adatom on graphene and one of the hydrogen atoms of chitosan is only 2.05 Å, and that between the H atom of the OH group on graphene and the closest oxygen atom of chitosan is only 2.09 Å. These close distances lead to strong charge redistributions and transfers between the graphene adsorbates and chitosan, as shown in Figure 6. The calculated value of 0.67 eV for the adsorption energy to chitosan on GO compares well with the previously reported value of 0.53 eV obtained with the B3LYP functional.^22^ In the case of GOH, a contribution to the binding energy may also come from the hydrogen bond between the OH group on graphene and that on chitosan. The stronger binding in the case of OH functionalization of graphene is in agreement with the result of a previous paper.^28^ The increase in binding energy with oxygen surface functionalization is a promising result for application to batteries and is in agreement with the mechanical strengthening observed when chitosan is combined with reduced graphene oxides. As a binder, it would render the electrode material less prone to cracks.

The effect of SEI component LiF on the binding energy between chitosan and graphene is significant. The binding energy of chitosan in GLiF is the highest. In this case, LiF detaches spontaneously from the graphene sheet, and the Li atom attaches to the O atom of the primary hydroxyl group and to the O atom of the chitosan ring. If we calculate the binding energy of the chitosan–LiF system to pure graphene, we find that the binding energy is 0.42 eV. This value is obtained using the expression: −[*E*(GLiF + chitosan) – *E*(Chitosan + LiF) – *E*(graphene)] where the binding energy is calculated as −[*E*(GLiF + chitosan) – *E*(Chitosan) – *E*(GLiF)]. The lower value of 0.42 eV represents the difference between the binding energies of LiF to graphene or to chitosan. The result shows that LiF has a larger binding energy to chitosan than to graphene. Thus, if it is present in the environment, it would attach to chitosan and not to graphene. These findings point to the fact that coupling between chitosan and Li could potentially facilitate the Li diffusion into graphite, which is also supported by the work of Zhou et al.^58^

In order to extract the contribution to the binding energy trends shown in Figure 5 due to a pure electrostatic interaction, we used a simple model. We calculated the Coulombic interaction between graphene and chitosan based on the total charge transferred between chitosan and graphene (calculated with the Bader charge analysis) and the chitosan–graphene distances at 0 K reported in Figure 5b. We used these closest distances between chitosan and graphene because the charge transfer is the largest between closer atoms (see Figure 4c). As seen in the graph, the trend of the DFT-calculated binding energies at 0 K is roughly reproduced by the Coulomb energies. The model explains, for example, that the binding energy between chitosan and GV is higher compared to that between chitosan and pure graphene (G) because the distance between chitosan and GV is smaller and the charge transfer is higher. The largest discrepancies between the DFT-calculated binding energies and the model are those related to GO and GOH. A close inspection indicates that the electronic charge gained by the oxygen atom of graphene in GO and GOH is high, −0.83 and −0.5 e, respectively. The strong interaction between the negatively charged O atom on graphene and the positively charged H atoms of chitosan due to the hydrogen bond discussed above is missing in the Coulombic model but contributes greatly to the binding energy.

We analyze the charge redistribution occurring when chitosan adsorbs on graphene in Figure 6. In the cases of GV, GB, and GN, where the point defect is located below the chitosan amino group, the charge redistribution is similar and larger for GV. In the case of GO, there is charge depletion in the bond between the O atom and C atoms of graphene and charge accumulation between the O atom and chitosan. In the case of GOH, the H atom of the OH group on graphene loses electrons, while the O atom of the same group gains electrons upon interaction with chitosan. The net charge transfer is inverted from chitosan to graphene. In the GLiF case, after LiF attachment to chitosan, the bond between Li and F loses electrons, weakening the bond, while the O and F atoms gain electrons.

We also calculated the planar-averaged charge difference along the out-of-plane direction for all optimized configurations (Figure 7). First, we see that in the cases of G, GV, GB, and GN, the distribution shows a net negative charge difference (charge depletion) on the graphene layer and a more positive charge difference on chitosan (charge accumulation, in agreement with the calculated charge transfers, reported in Figure 5). The surplus of positive charge difference (more electrons) in the region between the graphene layer and the chitosan monomer shows the binding between them in agreement with the calculated positive binding energies. Larger the electronic charge accumulation in the region between graphene and chitosan, the stronger the binding. The redistribution of the charge on chitosan is similar in all cases but is reversed in the GOH case. Of particular interest is the negative peak in the region between graphene and chitosan, close to chitosan, that is evident mostly in GV, GB, and GOH. This region corresponds to the electronic charge depletion at the chitosan hydrogen atoms. In the cases of GV and GB, the charge redistributions are very similar to subsequent layers of charge depletion and accumulation forming dipolar layers oriented in the direction normal to graphene. We notice that GV and GB are indeed the only cases where the graphene layer has a lower number of electrons compared with that of pristine graphene.

**Figure 7:**
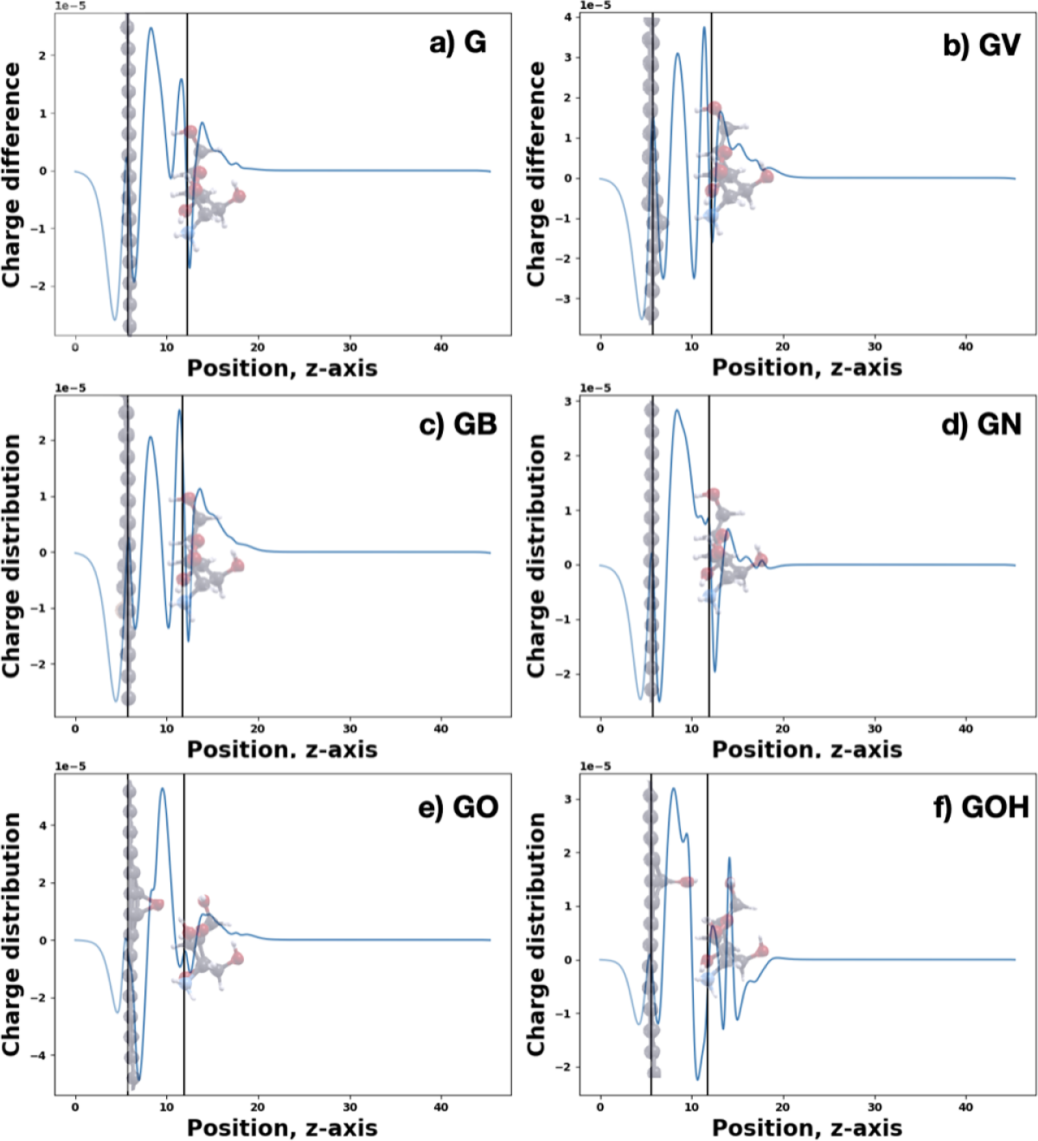
Plane-averaged charge difference distribution upon adsorption of chitosan on pure and defective graphene (a–f).

To link the charge transfers and redistribution to some energy scale, we show in Figure 8 the LDOS of all structures. In Figure 8a,b, we compare the total LDOS of isolated graphene with the LODS of chitosan adsorbed on graphene. The LDOS of the C atoms of graphene in 8 (b) (yellow lines) comprises only the contribution of C p orbitals since s orbitals do not contribute substantially in the energy range around the Fermi level. The comparison confirms that the small charge transfer from graphene to chitosan does not substantially change the distribution in energy of the graphene and chitosan states. This fact confirms the physisorption of chitosan on graphene and lack of state hybridization, which is a beneficial result, favoring chitosan as a binder for Li–ion battery anodes, considering that hybridization of the defect states with graphene can impede the superior electronic conductivity of graphene by acting as trapping centers. Moreover, strong covalent bonds could reduce the flexibility of the structure, which can result in cracks due to volume changes with lithiation. In all LDOS shown in the figure, we can see that the behavior at the Fermi level is decided by the electronic states of graphene. Thus, we see that the simple adsorption of chitosan on graphene does not affect the graphene electronic conductivity properties. Graphene remains a semimetal with Dirac cones at the *K* points. By comparing Figure 8b,c, we observe a shift of the Fermi level to lower energies (relative to the valence edge of chitosan) and the formation of peaks below and above the Fermi level in the p orbital energy distribution of graphene C. The shift to lower energy and the formation of the peak just above the Fermi level can be explained with the change of p bonding states of graphene carbons to a no-bonding state caused by the presence of the vacancy. The presence of the peaks below and above the Fermi level can have a strong effect on the electronic behavior since these states are mostly localized states at the vacancy and will work as trapping states on the electron motion.^59^ The interaction between chitosan and GV leads to a very small change in chitosan LDOS at about −1.2 eV. A similar shift toward lower energies of the Fermi level is also present in the LDOS of chitosan on GB (relative to the chitosan LDOS edge) as shown in Figure 8d. These shifts at lower energies are related to the missing electron(s) in graphene after the extraction of the carbon atom (GV) or the boron to carbon substitution (GB). The incomplete orbital filling also introduces similar peaks in the graphene carbon LDOS below the Fermi level as for GV. Relative to pure graphene, now there are few states at the Fermi level and partially occupied hole bands, which could produce electric current and intraband absorption at very low excitation energies. GN (Figure 8e) has an extra electron with respect to G; thus, the Fermi level moves to higher energies, and a half-occupied peak due to the graphene carbon states appears above the Fermi level. In this case, the C density of states at the Fermi level is quite high so we expect a higher conductivity due to the extra N-related almost free electrons. On GO and GOH (Figure 8f,g), electrons are transferred from the graphene carbon atoms to the oxygen adsorbate. This electron transfer creates new localized states that show as peaks at the Fermi level. These peaks are more spread in their energies for GO than for GOH, probably because in the latter case, O is less electronegative having already acquired an electron from H. These localized states at the Fermi level due to the isolated adsorbates would impede electronic transport acting as traps. Finally, in the case of GLiF, since LiF detaches from the graphene sheet and attaches to chitosan, the LDOS of graphene is recovered around the Fermi level.

**Figure 8:**
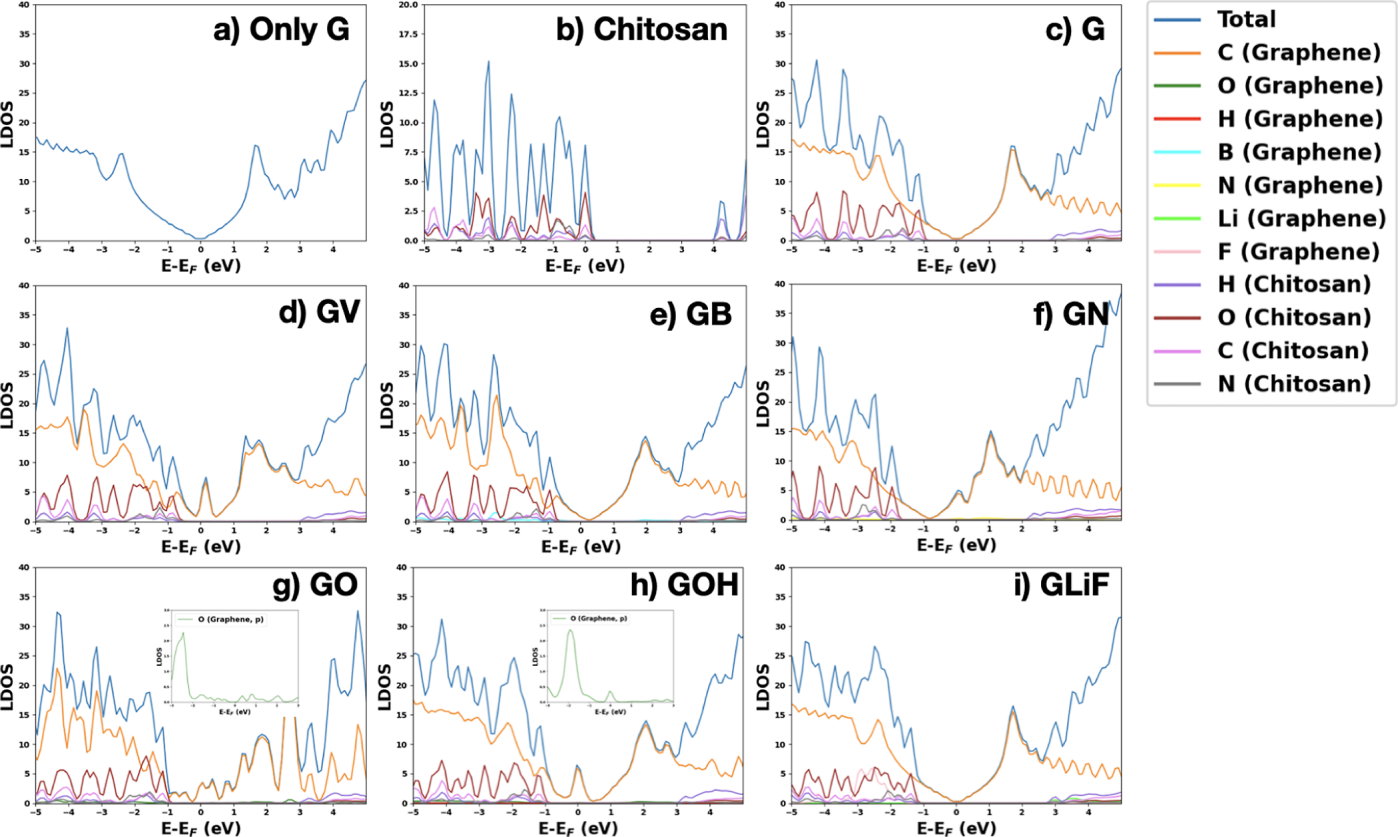
LDOS of graphene (a), isolated chitosan (b), chitosan adsorbed on pure, and defective graphene (c–i). For graphene C atoms, only the p orbital contribution to the LDOS is presented.

In the case of PVDF, the dependence of the binding energy on the molecular orientation relative to the graphene sheet is reported in Figure 9a. We can see that PVDF has generally a larger binding energy than chitosan. The most stable configuration is Ori.5, with the larger number of hydrogen atoms facing graphene. In the figure, the equilibrium distances are also reported. We notice that the presence of defects in graphene has a minimal effect on the values of the binding energies, as seen in Figure 9b. Even when the oxygen atom and the hydroxyl molecule are adsorbed on graphene, the binding energy does not increase much. Only the presence of LiF increases the binding energy in a significant way. LiF breaks down and the F atom of LiF replaces one of the H atoms of PVDF while the detached H atom binds to the graphene surface with the Li atom still adsorbed to the surface of graphene.

**Figure 9:**
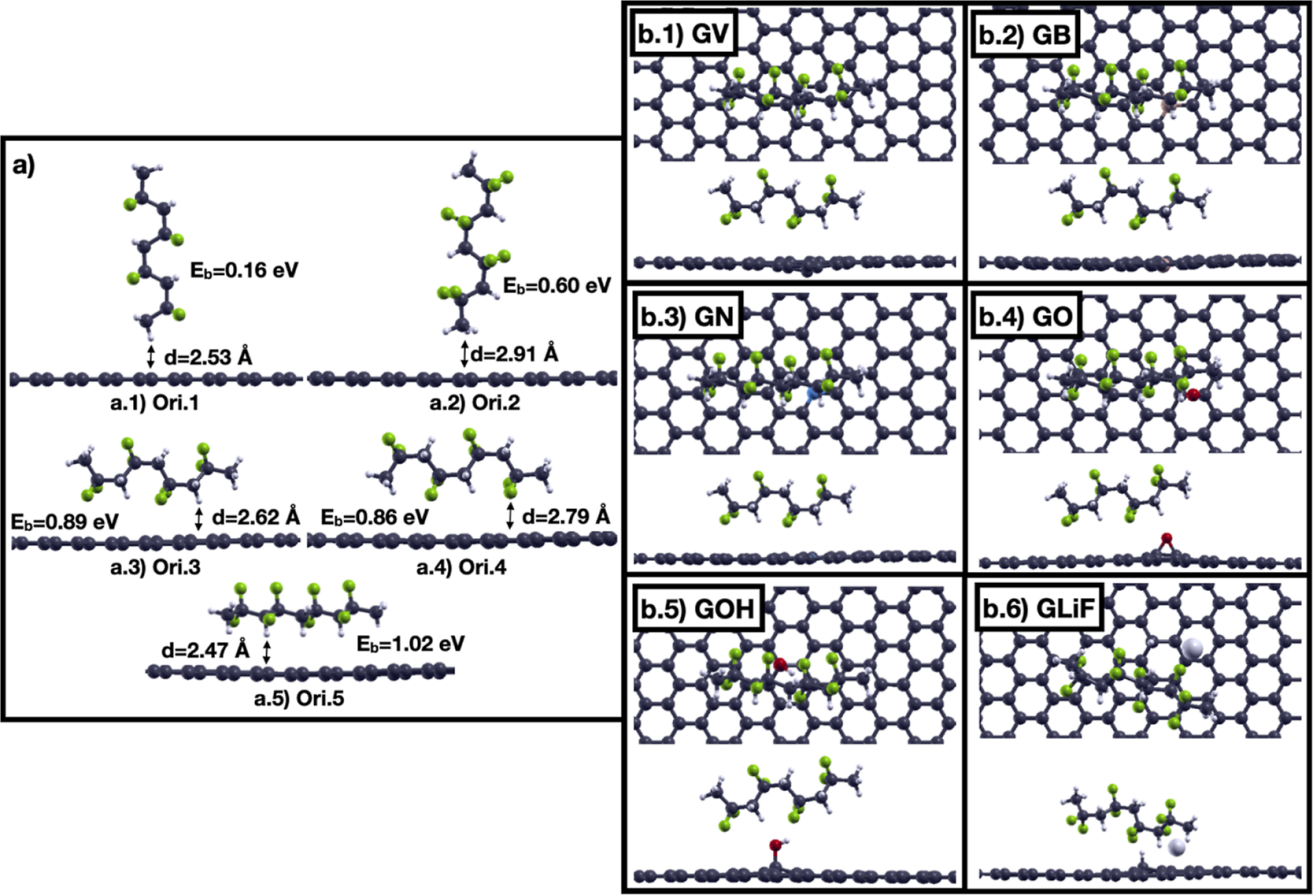
(a) Binding energies (*E*_b_) and graphene–PVDF distances for five different orientations of PVDF relative to graphene, (b) optimized structures of PVDF adsorbed on defective and functionalized graphene. Color codes: C: dark gray, B: orange, N: blue, O: red, F: green, H: small white, Li: large light gray.

Thus, our results show that the properties of chitosan can be effectively changed with functionalization of the graphene surface and, possibly, of chitosan itself, while PVDF shows less flexibility, although PVDF has a higher binding energy compared to chitosan at 0 K, which can be related to a larger charge transfer from pure graphene (−0.048 e).^23–29,34^ The changes with graphene functionalization are smaller (in percentage) for PVDF due to the F ions having very large electronegativity, which influences the structure of the PVDF system and PVDF having a larger symmetry. This characteristic of chitosan makes it promising for a number of applications, including as a binder or electrolyte in Li–ion batteries.

Finally, we investigate environmental effects relevant to battery operation, namely, lithiation, pH, and temperature, in the following sections.

### 3.3 Multilayer Graphene and Lithiation

In this section, we discuss the dependence of the chitosan binding energy on the number of graphene layers (Figure 10a) and on the Li intercalation (LiC_6_) (Figure 10b). We used for LiC_6_ the lowest energy AA stacking of the C layers, as opposed to the AB stacking of the unlithiated multilayer graphene.^60^ The optimized in-plane lattice constants of unlithiated and fully lithiated (LiC_6_) multilayer graphene (without chitosan) are found to be 4.27 and 4.30 Å, respectively. In the case of LiC_6,_ our calculated lattice parameter is in very good agreement with the experimental values ranging between 4.290 and 4.316 Å^61^ and with the other theoretical results obtained with other van der Waals functionals.^61–63^ The in-plane C–C distance is only slightly increased with lithiation, in agreement with previous studies. The in-plane lattice parameter does not change after chitosan adsorption. As for the interlayer distances, lithiation increases it by 13.5%, while chitosan adsorption decreases the interlayer distance between the first two layers only by 0.9%. The chitosan distance to graphene, where the perpendicular distance of the closest H atom to C atom of graphene is considered, increases from the monolayer to bilayer graphene from 2.51 to 2.56 Å, while it is slightly reduced from 2.56 to 2.50 Å with lithiation. The orientation and geometry of chitosan do not change.

**Figure 10:**
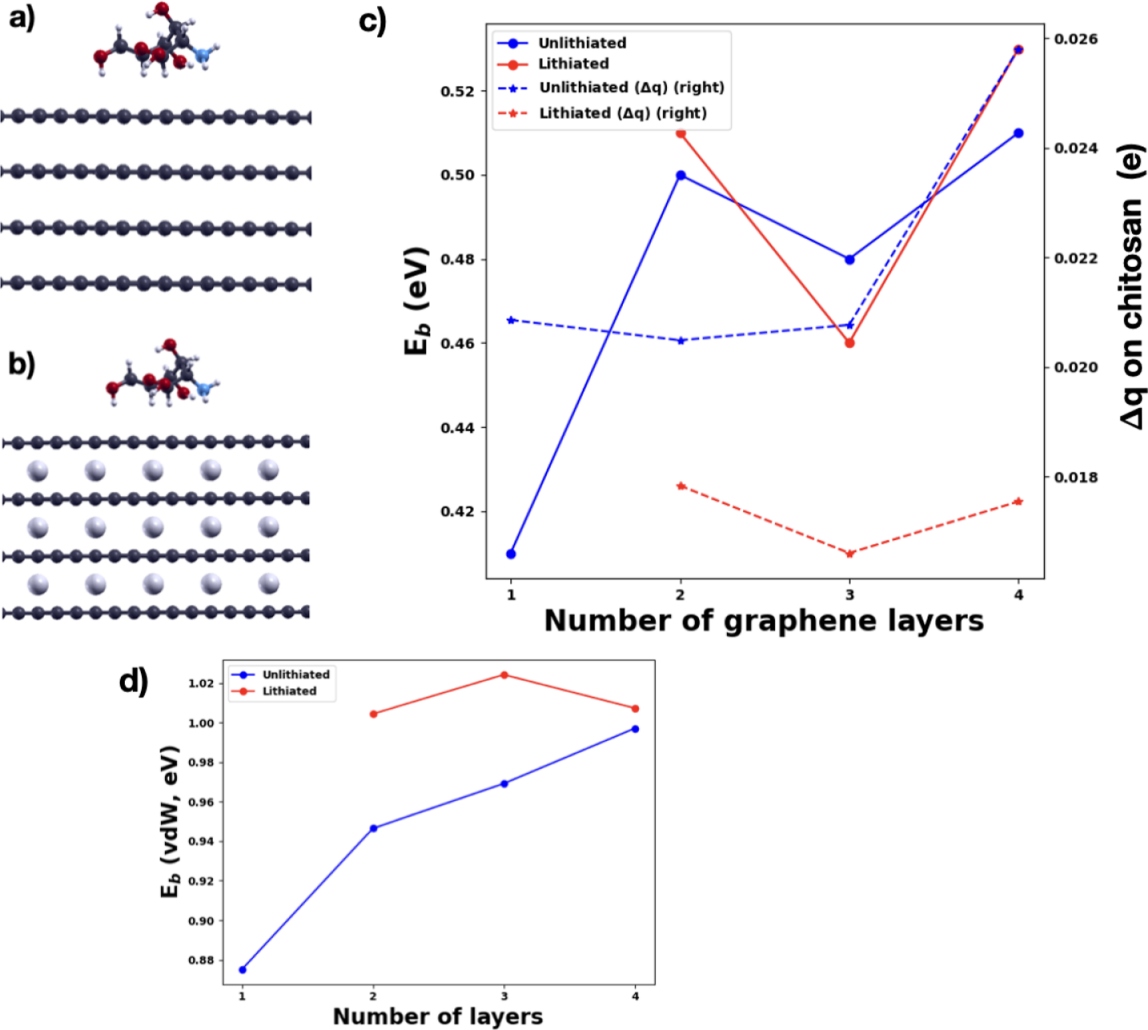
Four layers of graphene: (a) unlithiated, (b) fully lithiated, (c) binding energy and Bader charge transfers to chitosan as a function of graphene layers, and­(d) van der Waals interaction contribution to the chitosan binding energy as a function of graphene layers. Color codes: C: dark gray, O: red, N: blue, H: small white, Li: large light gray.

Increasing the number of graphene layers, the binding energy increases with a zigzag behavior (Figure [Fig fig1]0c). This zigzag behavior versus the odd or even number of layers in slab calculations has been reported previously in the literature for surface energies, band gaps, and adsorption energies.^64–67^ Eventually, these quantities will converge by increasing the number of layers in the slab. The zigzag trend has been ascribed to a number of different effects, even computational artifacts, such as the Brillouin zone sampling. However, it is clear from Figure [Fig fig1]0c that the increase of the binding energy from monolayer to multilayer graphene cannot be a computational artifact. We found that this behavior correlates well with the larger charge transfer in multilayer graphene than in monolayer graphene (see Figure S4a). These charge transfers create more and larger dipoles within the system, which increase the van der Waals interaction contribution to the chitosan binding energy, as shown in Figure [Fig fig1]0c. So, our results show that the van der Waals interaction plays a dominant role in determining the higher chitosan binding energy to multilayer graphene. This effect has already been found in the literature^68^ and was used to explain the increase of the interlayer binding between graphene layers with the number of layers. This interlayer binding is evaluated through the interlayer binding energy defined in eq 5. Our calculated values for the interlayer binding energies are 56.7 meV/atom in 2G, 57.6 meV/atom in 3G, and 58.6 meV/atom in 4G.

From Figure 10c, we also see that the charges transferred from the graphene systems to chitosan are not correlated to the binding energy strengths. While the chitosan binding energies to the fully lithiated multilayers are similar to those to the unlithiated ones, the charge transfers to chitosan from the lithiated multilayers are much smaller. As in the case of unlithiated multilayer graphene, lithiation leads to an increase of the van der Waals interaction strength and of the charge dipoles on the two sides of the graphene layers (see Figures 10d and S4).

The effects of lithiation in graphene/graphite have been extensively studied in the literature. Density functional studies found that lithium ions are completely ionized and their 2s electrons are transferred to empty pz carbon orbitals.^62^ These electrons go to strengthen the in-plane charge between the C–C bonds.^69^ Our calculated orbital projected density of states shown in Figure 11a,b agree with those published and support this affirmation. We show in Figure 11c,d the charge density redistribution of bilayer graphene caused by the insertion of Li atoms. The charge density increases along the in-plane C–C bonds, strengthening the in-plane σ bonds. The electronic charge of the p_*z*_ orbitals perpendicular to the layer (and therefore extending toward chitosan) is depleted. This charge depletion explains the lower transferred charge from graphene to chitosan. The binding energy increases with lithiation despite relatively lower charge transfer, and this can be understood with the increased polarization of the closest graphene layer to chitosan and also chitosan itself (see Figure S4a). These results are promising in terms of the electrochemical performance of the graphite anode where chitosan is used as a binder.

**Figure 11:**
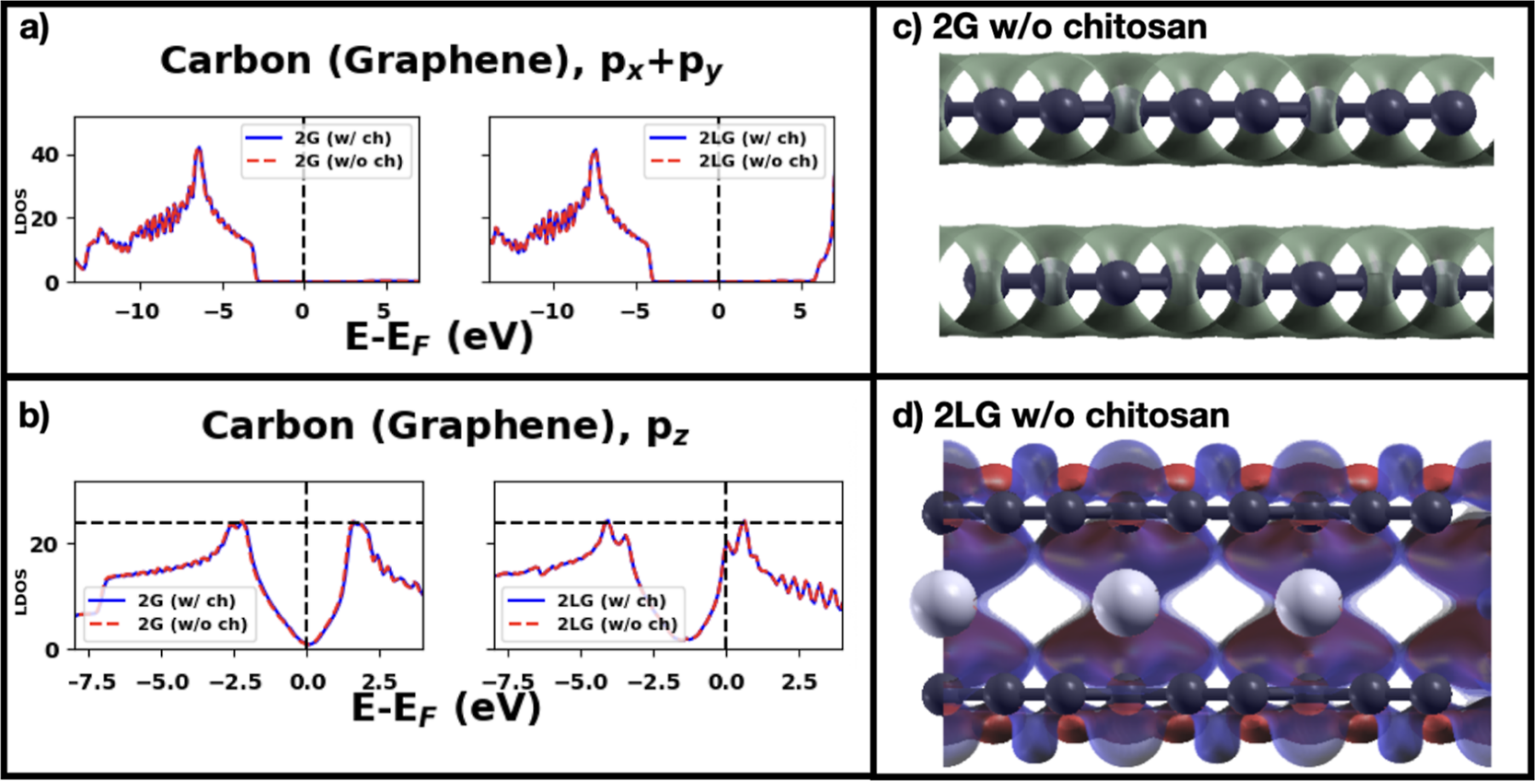
LDOS of the C atoms of graphene for 2G and 2LG: (a) p_*x*_ + p_*y*_ orbitals, (b) p_*z*_ orbital, (c) charge density distribution of the isolated bilayer graphene, and (d) charge density difference upon Li intercalation of the isolated bilayer graphene. Isovalue in (a,b,d) is 0.0003, while it is 0.03 in (c). Color codes: C: dark gray, O: red, N: blue, H: small white, Li: large light gray. In the charge density difference plot of the lithiated bilayer graphene (d), the blue areas represent charge depletion and the red areas represent charge accumulation.

### 3.4 pH of the Solution

As we discussed in the introduction, it was found experimentally that the pH level affects the chitosan properties. Acidic and basic environments contain H^+^ and OH^–^ groups in solution, respectively, and here, we assume that these groups attach to the chitosan monomer. Therefore, we modeled the effects of the environmental pH on chitosan by substituting a H atom in the NH_2_ group with H^+^ and OH^–^, where the first substitution is used to model the acidic environment and the second to model a basic environment. The substitution of the H atom with H^+^ is not a proper protonation of the chitosan monomer, whose effect on chitosan adhesion will be studied later. It provides, however, a good indication. The binding energies at infinite distance resulting from the fit of the calculated binding energies at varying unit cell dimensions are shown in Figure 12, while the fitted curves are given in Figure S5. We see that both the acidic and basic environments greatly increase the chitosan binding to graphene (>4 eV). This result is important for applications of chitosan as a binder in Li–ion batteries since chitosan needs to be solved in diluted acidic solutions, and some acid functional groups remain attached to it after drying. We can see that the binding energy slightly increases with an increasing number of layers for both situations. Lithiation significantly increases the binding energy in the acidic environment while decreasing it in the basic environment (see 2LG case in Figure 12).

**Figure 12:**
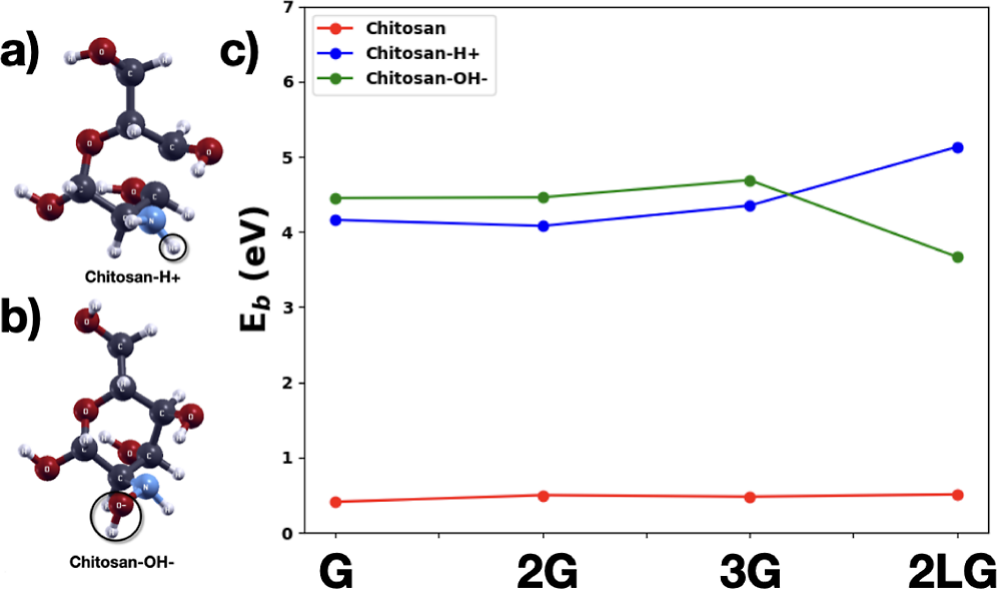
Structures of (a) chitosan-H+, (b) chitosan-OH^–^, and (c) binding energies of chitosan-H+, chitosan-OH^–^, and neutral chitosan as a function of unlithiated and lithiated multilayer graphene. Color codes: C: dark gray, O: red, N: blue, H: small white.

We have shown that in the neutral state a charge transfer of 0.021 *e* occurs from graphene to chitosan; therefore, chitosan is in a negative state of charge and graphene is in a positive state of charge. The calculated charge transfer in the case of the positively charged hydrogen atom is 0.982 *e* (almost 1 electron) from graphene to chitosan. The transferred charge increases to 0.989 *e* for bilayer graphene and to 0.992 *e* for trilayer graphene. In the case of OH^–^, the charge transfer of 0.936 *e* occurs from chitosan to graphene. In both cases, chitosan tends to become neutral while the graphene layers acquire or lose electronic charge. These relevant charge transfers lead to a much stronger binding between chitosan and the charged graphene layers through strong electrostatic interaction, which could potentially lead to self-healing property supported by the work of Peng et al.^70^

The presence of OH^–^ leads to an additional attraction between OH^–^ since without charging graphene would be slightly positively charged when exposed to chitosan. This can explain the slightly higher binding energy for the basic environment.

As seen in the previous section, the Li atoms inserted between the graphene layers transfer their electrons to graphene, making the carbon layers negatively charged. As a consequence, the H^+^ ion interacts attractively with the negatively charge graphene increasing the binding energy of the H^+^ case, while the OH^–^ ion interacts repulsively reducing the binding energy.

### 3.5 Effect of Room Temperature on the Binding Energies

So far, we have discussed the binding energies obtained by force optimization calculations starting from an initial configuration at 0 K. However, the final structures obtained this way may be in local energy minima. In order to find energetically more favorable orientations, we performed AIMD at a temperature of 300 K. In Figure 13, we report the binding energies obtained at 300 K.

**Figure 13:**
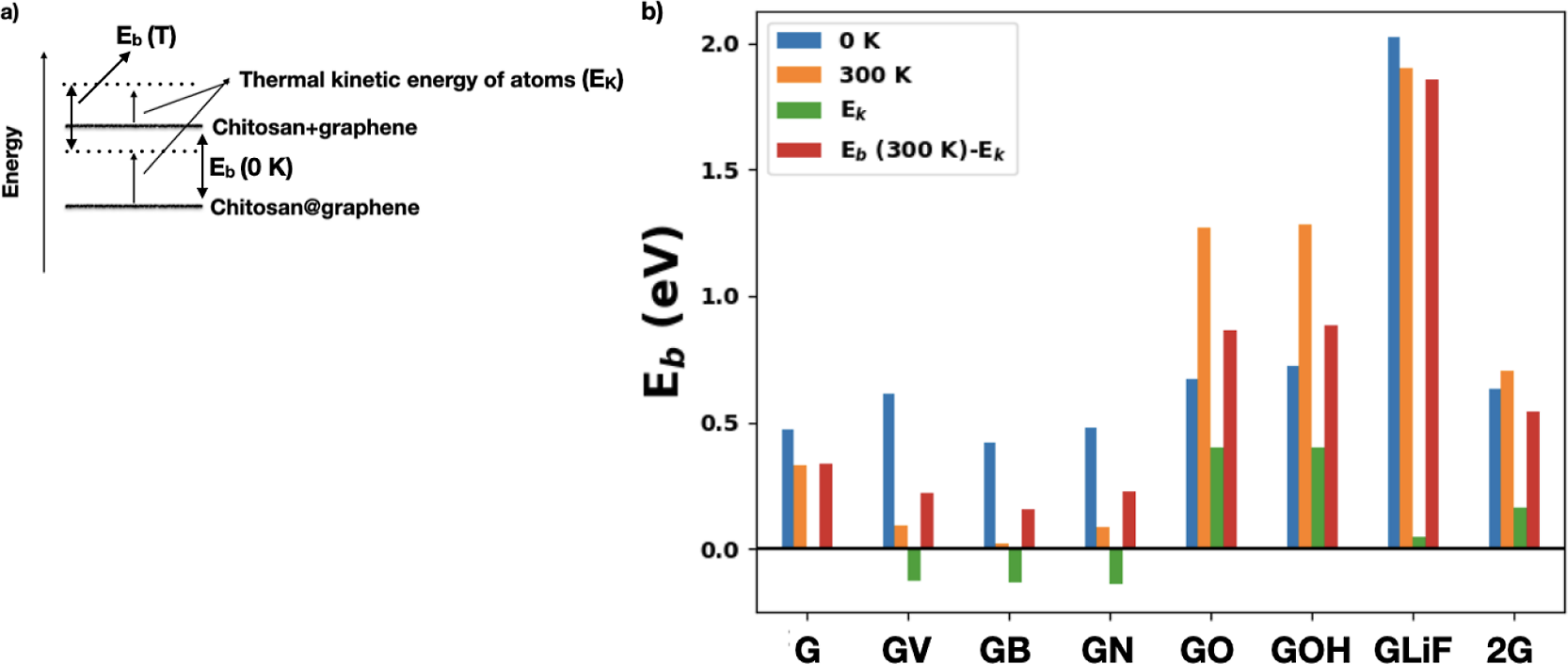
(a) Schematic representation of the effect of the thermal kinetic energy (*E*_K_) on the chitosan binding energies, (b) binding energies at 0 and 300 K where also the contribution of the kinetic energies *E*_K_ to the binding energies is shown.

In Figure 13, we see that chitosan has a lower binding to pure graphene (G), to the defective GV, GB, GN, and to GLiF at 300 K compared to that of 0 K. This means that the chitosan orientation and configuration obtained at 0 K are already in an energetic minimum. The influence of the positive kinetic energies of the atoms (see Figure [Fig fig1]3 a for explanation) contributes to decrease the binding and leads to a configuration of the system, which is, on average, less stable.

Figure 14 shows the final structures (at 3 ps) at 300 K compared with those obtained at 0 K. In the case of pristine graphene, the hydrogen atom of one of the terminal groups turns toward the graphene sheet and the OH molecule of the primary hydroxyl group orients itself so as to stay parallel to the graphene surface at 300 K. In the case of GV, chitosan orientation does not change at 300 K relative to 0 K, apart from an increase of the distance of the amino group to graphene. In the case of GB, chitosan rotates so that one of the terminal groups turns toward the surface and the other terminal group on the other side of chitosan turns away from the graphene surface. In the case of GN, the orientation of chitosan remains almost constant at 300 K where there is only a rotation of the OH group in the primary hydroxyl group. In the case of GLiF, the Li atom of the LiF molecule, which is attached to chitosan, moves closer to graphene, while the opposite side of chitosan moves further away.

**Figure 14:**
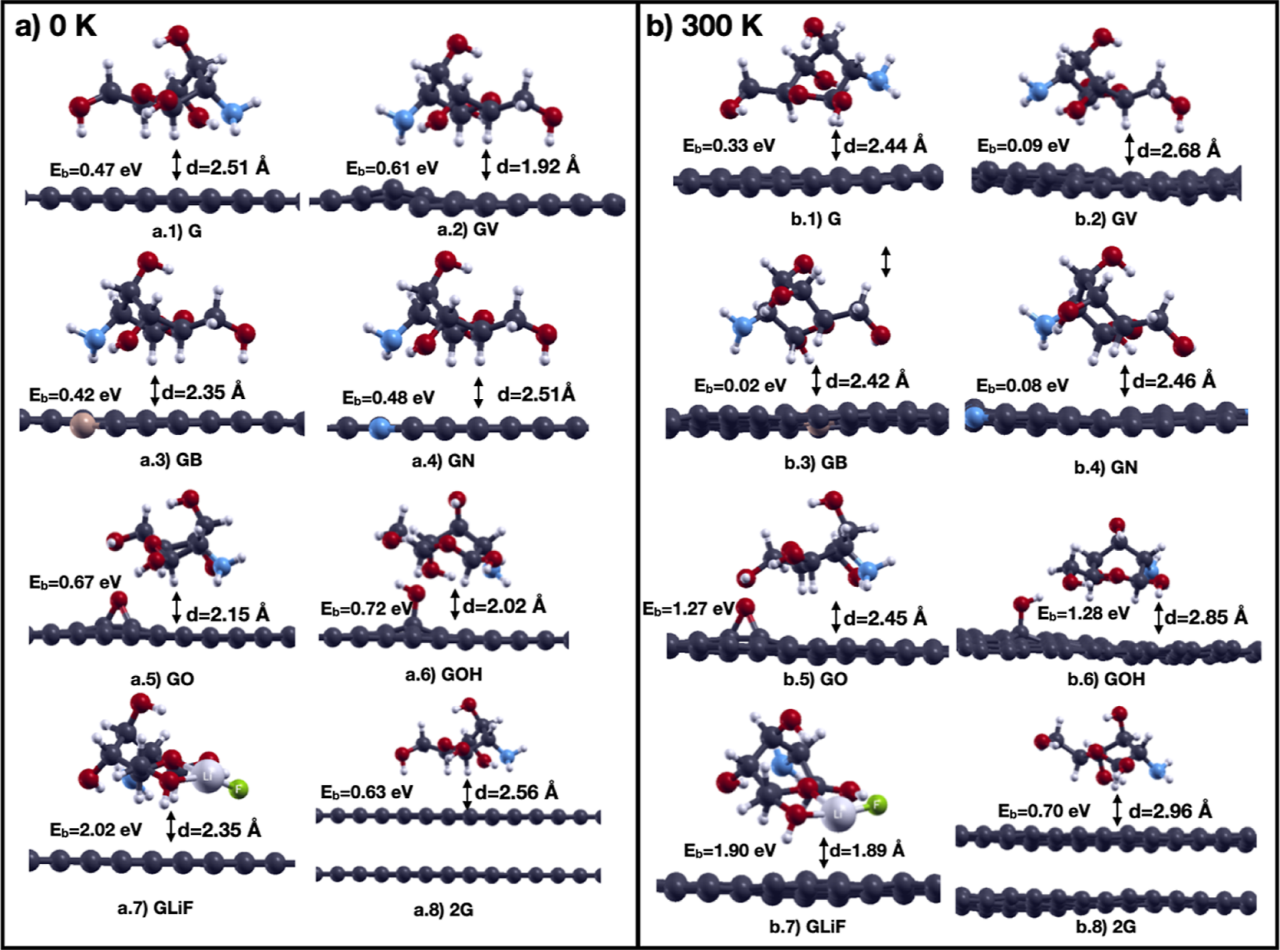
Comparison of the structures obtained at (a) 0 K with force optimization and (b) 300 K with ab initio MD. Color codes: C: dark gray, O: red, B: orange, N: blue, H: small white, Li: large light gray, F: green.

On the other hand, the binding of chitosan to GO and GOH is greatly improved at 300 K compared to that at 0 K. The reason that the binding energy on GO is higher than on pure graphene at 300 K compared to that at 0 K is because the distance between the O atom adsorbed on graphene to the H atom of the primary hydroxyl group of chitosan is greatly reduced from 3.27 to 2.63 Å, while its distance to the closest H atom slightly increases (from 2.07 to 2.28 Å). As a consequence, the O atom on graphene is interacting with two H atoms of chitosan instead of only one. Similarly, in the case of GOH, the distance between H of OH on graphene to the O of the primary hydroxyl group of chitosan is reduced at 300 K compared to that at 0 K (from 2.09 to 2.02 Å), therefore increasing the binding energy. Therefore, the orientations of chitosan on GO and GOH obtained at 300 K are energetically more favorable (see also Figure 13 where we show the binding energies stripped of the positive thermal kinetic energy contribution). These new structures of GO and GOH have chitosan bonded to graphene almost as strongly as that of PVDF.

We see that at 300 K chitosan has a significantly larger binding energy (0.70 eV) to two layers of graphene (2G) than to a single layer (0.33 eV) due to the larger van der Waals interaction strength (see Figure 10), and its binding to 2G at 300 K is also larger than the binding to 2G at 0 K (0.63 eV). This difference becomes larger at 300 K temperature (orange bars in Figure 13) due to the higher contribution from the kinetic energy of the combined system, chitosan, and two layers of graphene. As for the structural changes, the monomer of chitosan on bilayer graphene at 300 K tilts in such a way that the primary hydroxyl group moves away from the surface (see Figure 14). The temperature and the thickness of the graphene film have thus important effects on the structural configuration, distance, and binding energy of chitosan. Our analysis will form the baseline for the understanding of the binding of more complicated structures of chitosan oligomers and polymers to graphene and graphite.

As a result, the effect of the temperature is in general to improve the binding energy of chitosan to graphene; however, the nature of the interaction remains to be a combination of electrostatic and van der Waals interactions. These findings point in the direction that at room temperature, still the state hybridization is lacking and therefore should preserve the superior electronic properties of graphene, thus favoring chitosan as a suitable binder for Li–ion battery anode graphite.

## 4 Conclusions

In conclusion, we studied the properties of chitosan to both pure and defective graphene. We also analyzed the impact on the binding energy of chitosan of multiple layers of graphene and of the presence of lithium between the layers. We have compared the calculated binding energies with those of the binder PVDF, commonly employed together with the active graphite anode material in Li–ion batteries. Our studies have been conducted using accurate DFT calculations that take into account the long-range van der Waals interactions. We have found that chitosan binds to graphene less strongly than PVDF, a fact that could be related to a smaller charge transfer. Using the Bader charges approach, we have found that a small charge transfer of 0.021 *e* occurs from graphene to chitosan. The small value of the charge transfer together with the value of the equilibrium distance between chitosan and graphene suggests that the binding is dominated by the van der Waals interactions. The lack of state hybridization was confirmed by the calculated partial (projected on atoms) densities of states in the system. We found that the inclusion of defects in the graphene sheet such as single C vacancies, boron and nitrogen point substitutions, and the presence of adsorbates such as an oxygen atom or an OH dimer do not increase the binding energy of chitosan to graphene enough to make it larger than the binding energy of PVDF to pure graphene. However, the presence of these defects causes important changes in the binding properties, and, in particular, in the electronic charge redistribution and electronic states that we have studied through the detailed analysis of 3D and planar-averaged charge difference plots and of the atom-projected densities of states, respectively. While the presence of point defects introduces significant changes, the interaction between chitosan and graphene increases substantially in the presence of the OH and O and OH adsorbates. In the case of OH, the charge transfer is reversed, from chitosan to graphene, and the binding interaction is dominated by the hydrogen bond between the OH group on graphene and those on chitosan. We have also studied the case of a LiF molecule adsorbed on graphene since LiF is a salt often present in electrolytes. Structural optimization shows that LiF spontaneously detaches from graphene and attaches to the chitosan monomer. We have also investigated the interaction of chitosan with multiple layers (2 to 4) of graphene, pure or lithium intercalated, finding that the presence of lithium does not significantly affect the value of the binding energies. However, a significant decrease of the charges transferred from graphene to chitosan was found after lithium insertion, while the van der Waals contribution to the binding increases. The changes are explained in terms of charge redistribution within the graphene layers caused by the presence of lithium, leading to increased dipole and long-range dipolar interactions. The effect of the pH level of the solution is, instead, very significant. Low and high pH levels result in a strong binding of chitosan to graphene, which passes from 0.41 eV to more than 4 eV. The relevant result is that in both cases the charge initially located on the chitosan monomer due to the H^+^ and OH^–^ ions is almost totally transferred to graphene, increasing the binding. Ab initio MD simulations at 300 K reveal that the binding energy of chitosan to graphene oxide (i.e., with O and OH adsorbates) increases greatly and becomes slightly higher than the binding energy of PVDF at 0 K. Chitosan interacts differently from PVDF with the LiF electrolyte salt. In the case of chitosan, LiF transfers as a whole from graphene to chitosan, while in the case of PVDF, the F ion of LiF replaces one of the hydrogen atoms of PVDF with the detached H sticking then to the graphene sheet. In conclusion, our results show that chitosan has good adhesion properties to the graphene/graphite electrode and is easily modified for different applications through functionalization and changes in the environment. They provide a sound foundation for the design of optimal chitosan-based binders for electrodes in Li–ion batteries.

Importantly, while chitosan binds more weakly than PVDF to pristine graphene at 0 K, our results demonstrate that under realistic battery conditions—functionalized surfaces, finite temperature, and non-neutral pH—chitosan adhesion becomes comparable to or stronger than PVDF. This highlights chitosan’s potential as a tunable, environmentally benign binder rather than a direct drop-in replacement evaluated solely under idealized conditions.

## Supplementary Material



## Notes

Figure S1: Charge density difference of the defective graphene structures; Figure S2: benchmark calculations of benzene adsorption on graphene; Figure S3: Coulombic interaction calculated by taking the center of charge distance to graphene; Figure S4: plane-averaged charge distance plots for single and multilayer graphene including lithiation; and Figure S5: model function fitting of the charged cell calculations (PDF)
