# Supplementary material for: Understanding Binding of Chitosan to Graphene in Li–Ion Battery Anodes from First-Principles
Source: ACS Appl Energy Mater. 2026 Feb 4;9(4):2328–43. doi: 10.1021/acsaem.5c03926 (PMC12934541; doi:10.1021/acsaem.5c03926)
Supplement: Supplementary file 1 [file ae5c03926_si_001.pdf]

Supporting Information:

Understanding Binding of Chitosan to Graphene in Li-Ion Battery Anodes from First-Principles

Burak Ozdemir*[1] and Rita Magri[1,2,3]

[1]Dipartimento di Scienze Fisiche, Informatiche e Matematiche, Università di Modena e Reggio Emilia,Via Campi 213/A, 41125 Modena, Italy

[2]Centro S3, Istituto Nanoscienze-Consiglio Nazionale delle Ricerche (CNR-NANO),Via Campi 213/A, 41125 Modena, Italy

[3]Centro Interdipartimentale di Ricerca e per i Servizi nel settore della produzione, stoccaggio ed utilizzo dell'Idrogeno H2-MO.RE., Via Università 4, 41121 Modena, Italy

January 7, 2026

*email: burkzdemir@gmail.com

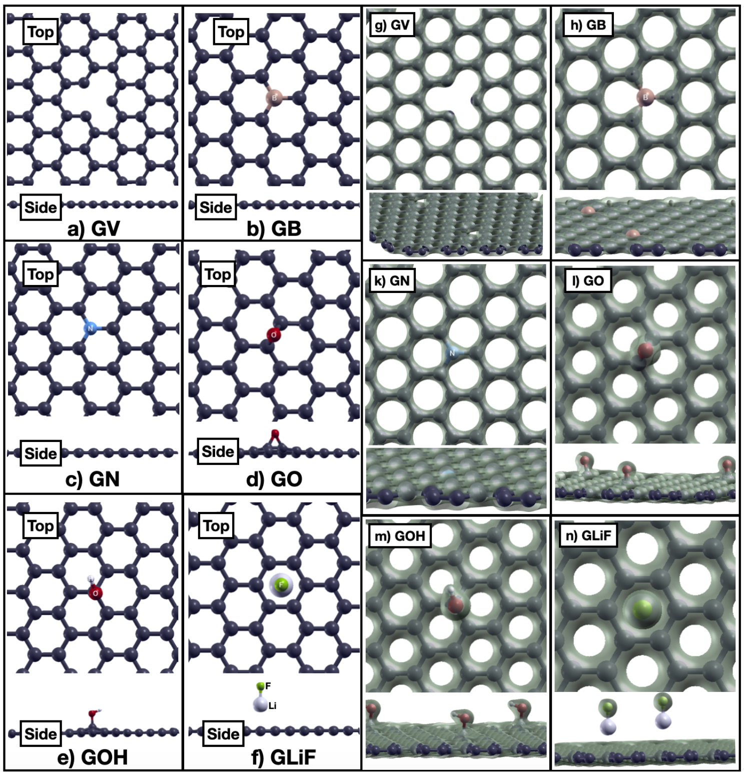

Figure S1: (a-f) Optimized structures of defective graphene and (g-n) their charge density distribution. Color codes; C: dark gray, B: orange, N: blue, O: red, H: small white, Li: large light gray, F: green.

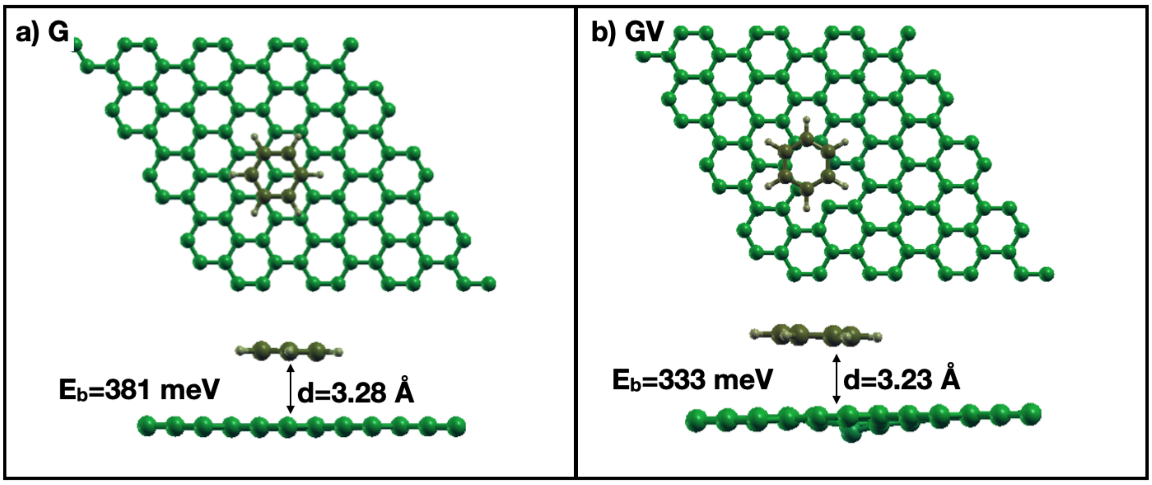

Figure S2: Benchmark calculations of benzene adsorption on (a) pure graphene and (b) graphene with single carbon vacancy

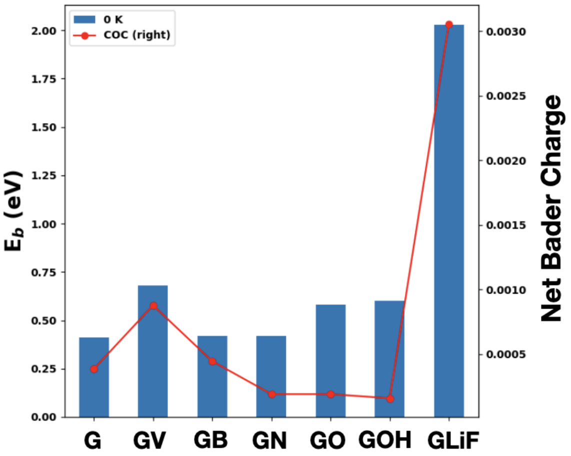

Figure S3: Comparison of binding energies to the Coulombic energies calculated by taking the center of charge (COC) distance to graphene

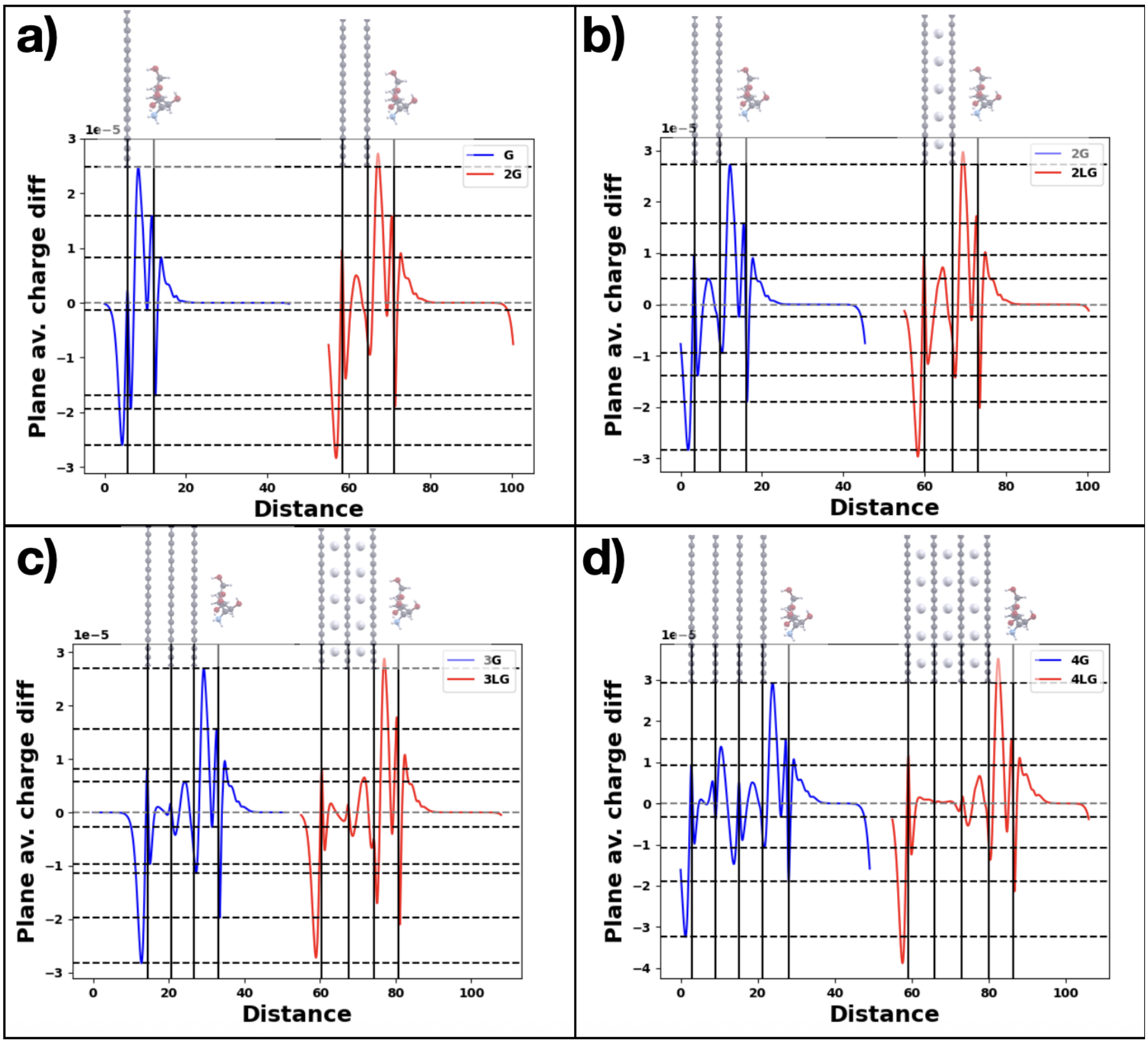

Figure S4: Plane averaged charge difference ($graphene/chitosan - graphene - chitosan$) in the out-of-plane direction for the (a) chitosan on monolayer graphene (G) and bilayer graphene (2G), (b) chitosan on bilayer graphene (2G) and lithiated bilayer graphene (2LG), (c) chitosan on trilayer graphene (3G) and chitosan on lithiated trilayer graphene (3LG), (d) chitosan on 4 layer graphene (4G) and chitosan on lithiated 4 layer graphene (4LG)

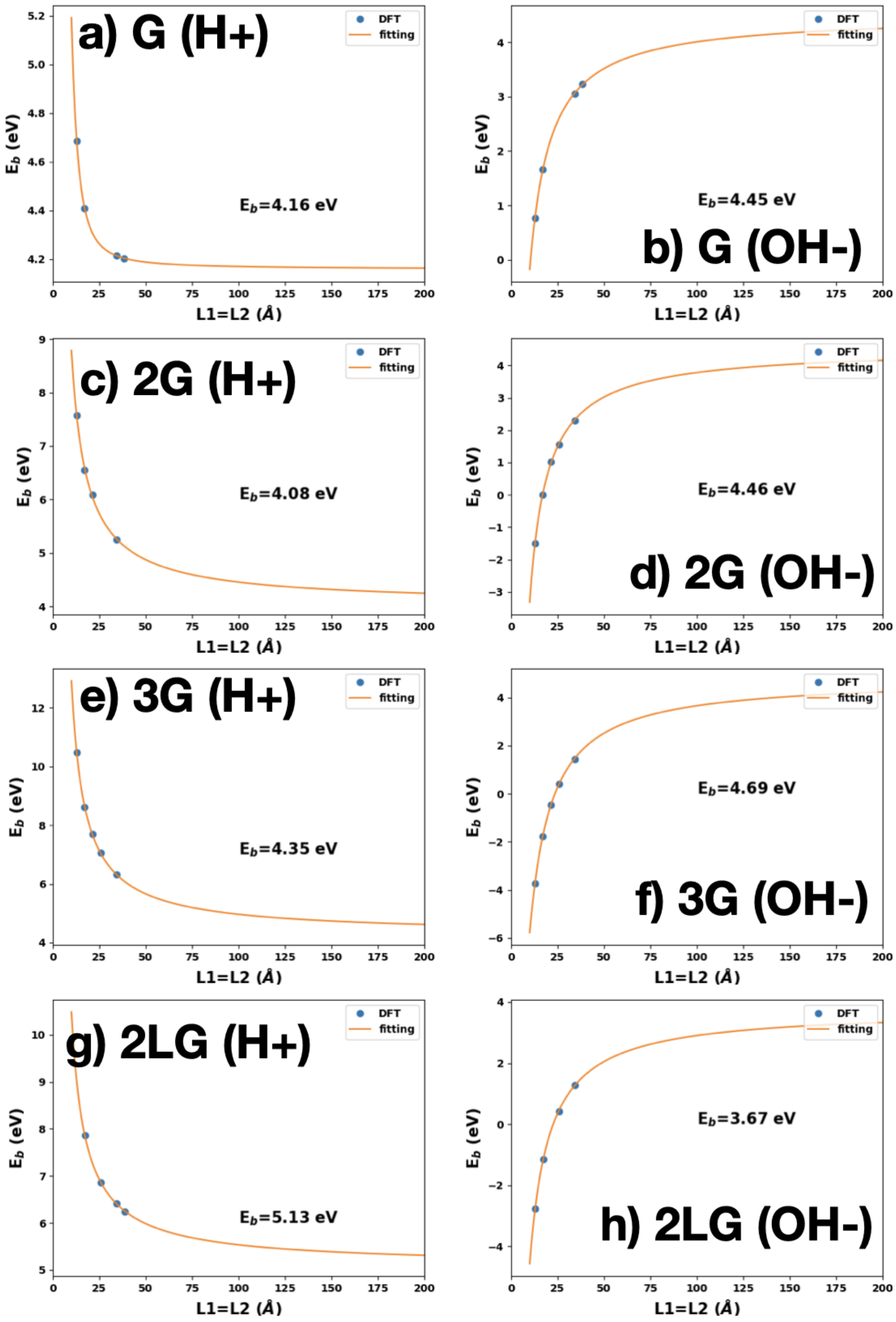

Figure S5: Model function fitting of the charged cell calculations where the calculated parameters of the model function are as follow; (a) a=4.158, b=1.167, c=930.280, (b) a=4.500, b=-49.292, c=258.356, (c) a=4.040, b=41.398, c=615.328, (d) a=4.538, b=-75.621, c=-297.486, (e) a=4.279, b=68.363, c=1826.651, (f) a=4.808, b=-114.860, c=922.812, (g) a=5.090, b=44.304, c=977.031, (h) a=3.755, b=-85.455, c=222.855
